# Medicinal Plants and Their Impact on the Gut Microbiome in Mental Health: A Systematic Review

**DOI:** 10.3390/nu14102111

**Published:** 2022-05-18

**Authors:** Eva-Maria Pferschy-Wenzig, Manuela R. Pausan, Karin Ardjomand-Woelkart, Stefanie Röck, Ramy M. Ammar, Olaf Kelber, Christine Moissl-Eichinger, Rudolf Bauer

**Affiliations:** 1 Department of Pharmacognosy, Institute of Pharmaceutical Sciences, University of Graz, Beethovenstraße 8, 8010 Graz, Austria; eva-maria.wenzig@uni-graz.at (E.-M.P.-W.); ka.woelkart@uni-graz.at (K.A.-W.); stefanie.roeck@edu.uni-graz.at (S.R.); 2 BioTechMed, Mozartgasse 12, 8010 Graz, Austria; christine.moissl-eichinger@medunigraz.at; 3 Phytomedicines Supply and Development Center, Bayer Consumer Health, Steigerwald Arzneimittelwerk GmbH, Havelstraße 5, 64295 Darmstadt, Germany; manuela.pausan@bayer.com (M.R.P.); ramy.ammar@bayer.com (R.M.A.); olaf.kelber@bayer.com (O.K.); 4 Department of Pharmacology and Toxicology, Faculty of Pharmacy, Kafrelsheikh University, Kafrelsheikh 33516, Egypt; 5 Diagnostic & Research Institute of Hygiene, Microbiology and Environmental Medicine, Medical University Graz, Neue Stiftingtalstraße 6 (MC1.B.)/III, 8010 Graz, Austria

**Keywords:** gut microbiome, gut microbiota, gut bacteria, phyto-psychobiotics, microbiome–gut–brain axis, gastrointestinal, mental health, medicinal plant, depression, anxiety, insomnia, cognitive impairment

## Abstract

Background: Various neurocognitive and mental health-related conditions have been associated with the gut microbiome, implicating a microbiome–gut–brain axis (MGBA). The aim of this systematic review was to identify, categorize, and review clinical evidence supporting medicinal plants for the treatment of mental disorders and studies on their interactions with the gut microbiota. Methods: This review included medicinal plants for which clinical studies on depression, sleeping disorders, anxiety, or cognitive dysfunction as well as scientific evidence of interaction with the gut microbiome were available. The studies were reported using the Preferred Reporting Items for Systematic Reviews and Meta-Analyses (PRISMA) statement. Results: Eighty-five studies met the inclusion criteria and covered thirty mental health-related medicinal plants with data on interaction with the gut microbiome. Conclusion: Only a few studies have been specifically designed to assess how herbal preparations affect MGBA-related targets or pathways. However, many studies provide hints of a possible interaction with the MGBA, such as an increased abundance of health-beneficial microorganisms, anti-inflammatory effects, or MGBA-related pathway effects by gut microbial metabolites. Data for *Panax ginseng*, *Schisandra chinensis*, and *Salvia rosmarinus* indicate that the interaction of their constituents with the gut microbiota could mediate mental health benefits. Studies specifically assessing the effects on MGBA-related pathways are still required for most medicinal plants.

## 1. Introduction

Stress, anxiety, mood disorders, sleep problems, and cognitive dysfunction are the most common mental health problems for which herbal products constitute a reasonable treatment option with minor side effects and low toxicity [1,2]. The pathogenesis of mental disorders is complex and generally thought to be linked to genetic, immune-related, humoral, neural, and environmental factors. However, various neurocognitive and mental health conditions have been strongly associated with imbalances in the gut microbiome composition, referred to as dysbiosis [3].

### 1.1. The Microbiome–Gut–Brain Axis (MGBA)

It is important to consider the symbiotic relationship between humans and their resident microbes when discussing the role of the gut–brain axis in behavior, health, and disease [4]. The sharp increase in various disease states in recent decades [5,6] could be explained, at least in part, by the changes in modern diets and lifestyles that have negatively impacted the composition and diversity of the human gut microbiome [7]. The gut microbiome could be the missing link in the conceptualization and treatment of psychological disorders [4]. The microbiome–gut–brain axis (MGBA) provides a network for signals from the brain to influence the motor, sensory, and secretory functions of the gut while simultaneously allowing signals and metabolites from the gut microbiome to influence brain development, biochemistry, function, and behavior [8,9,10].

**Figure 1:**
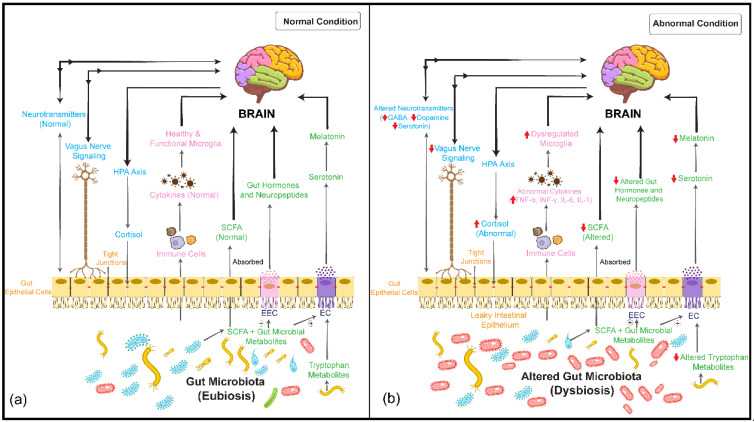
(**a**) Potential pathways involved in the communication between the gut microbiome and brain (microbiota–gut–brain axis, MGBA). (**b**) Alterations in gut microbiome (dysbiosis) and MGBA communication in neurodegenerative disorders. Gut microbiome–brain communication occurs mainly via three pathways: (1) neural (vagus and enteric nervous system, neurotransmitters, blue letters), (2) immune (cytokine balance and functional microglia, pink letters), and (3) humoral/metabolic (gut hormones, short-chain fatty acids (SCFAs), and neuropeptides, green letters). Neural communication is established via the vagus nerve and the hypothalamic–pituitary–adrenal (HPA) axis and systemic communication via the immune and humoral/metabolic pathways. In neurodegenerative disorders, the composition and activity of the normal gut microbiome are altered, leading to abnormal microbial metabolite profiles such as altered levels of neurotransmitters and SCFAs. The result is disruption of the neural, immune, and humoral/metabolic pathways and increased risk for disease progression [12,17,19]. The red arrows indicate alterations during dysbiosis (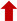 activation/upregulation, 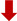 inhibition/downregulation). EC: enterochromaffin cell; EEC: enteroendocrine cell; SCFA: short-chain fatty acid; HPA: hypothalamus–pituitary–adrenal; TNF-α: tumor necrosis factor-α; INF-γ: interferon gamma; IL-6: interleukin-6; IL-1: interleukin-1; GABA: gamma-amino butyric acid. ⊕: stimulates/promotes.

The human intestinal microbiome predominantly consists of anaerobic bacteria, with the Firmicutes, Bacteroidetes, Proteobacteria, and Actinobacteria phyla constituting more than 90% of the total microbiota [11]. The gut microbiome is regarded as an important factor in bidirectional communication between the gut and the brain (gut–brain axis) [12,13,14]. This communication is based on several complex pathways that typically transmit sensory information from the gastrointestinal (GI) tract and subsequently convert it into hormonal, neural, and immunological signals. These signals further transmit information to the central nervous system (CNS) either individually or cooperatively [15]. Figure 1a shows how the gut microbiome can influence brain function via the gut–brain axis, thereby regulating behavior and psychological processes [12,16,17,18,19]. Microbiota–gut–brain interactions are thought to occur via three major pathways: (i) direct and indirect signaling via chemical transmitters such as microbial metabolites (e.g., short-chain fatty acids, or SCFAs), hormones, or neurotransmitters that can be either directly synthesized or modulated in their levels by gut microbiota; (ii) neural pathways, e.g., modulation of vagus nerve activity; and (iii) signaling within the immune system, e.g., microglia-mediated effects or effects of circulating cytokines that can modulate the activity of the hypothalamic–pituitary–adrenal (HPA) axis [11,12,16,20,21,22].

Regarding chemical signaling, microbiota-derived metabolites, and in particular SCFAs, are important signaling molecules. SCFAs are produced from carbohydrates by certain GI tract microorganisms and regularly absorbed by the colonocytes through H^+^-dependent or sodium-dependent monocarboxylate transporters. SCFAs are responsible for several local effects, including maintenance of intestinal barrier integrity, mucus production, and anti-inflammatory effects (lowering the risk for colorectal cancer). These beneficial effects of SCFAs, in turn, improve overall gut health [23]. Moreover, SCFAs exert substantial systemic hormone-like actions and show immunomodulatory and neuroactive properties [12,16,24]. SCFAs also control the production of gut peptides by enteroendocrine cells (EECs). These peptides modulate the gut–brain axis and stimulate the synthesis of gut-derived serotonin from enterochromaffin cells (ECs), subsequently influencing gut–brain hormonal communication [16]. Moreover, SCFAs can cross the blood–brain barrier (BBB) and control microglia homeostasis in the brain. This process is thought to be involved in proper brain development and in modulating behavior [14,16,25]. Butyrate, in particular, is of major interest given its ability to regulate gene transcription and has been shown to have an antidepressant effect in mice [26].

Apart from SCFAs, the gut microbiota can produce neurotransmitters in the epithelial lining and convert their precursors to active metabolites in the gut lumen [27]. Various GI bacteria such as *Lactobacillus* spp., *Bifidobacterium* spp., *Bacillus* spp., *Escherichia* spp., and *Saccharomyces* spp. are involved in the production of neurotransmitters such as gamma-aminobutyric acid (GABA), acetylcholine, noradrenaline, dopamine, and serotonin, and in the production of the serotonin precursor tryptophan. These neurotransmitters can, in turn, control neural signaling within the enteric nervous system (ENS) and eventually modulate brain function and behavior [12,16,28]. While neurotransmitters produced in the gut may not directly influence the brain as they do not pass through the BBB, they are able to influence the CNS through mechanisms including direct stimulation of the vagus nerve, as well as indirect circulatory and immune pathways [29]. Serotonin, the most well-studied neurotransmitter in relation to depressive illness, appears to be particularly susceptible to being influenced by the gut microbiome. A key study in 2009 revealed that the plasma serotonin levels of germ-free mice were almost three times less than those of conventional mice [30]. It was subsequently demonstrated that this differential serotonin level was secondary to the remarkable ability of gut microbes to directly promote the synthesis of serotonin from its amino acid precursor, tryptophan, in intestinal enterochromaffin cells [31]. Furthermore, the gut microbiome was also shown to influence serotoninergic levels in the hippocampus, an area of the brain which plays an important role in stress, anxiety, and depression [32]. Lyte [33] stated that probiotics function mechanistically as delivery vehicles for neuroactive compounds and that these probiotics have the potential to act as psychotropic agents.

The gut microbiota also seems to play a role in the production of brain-derived neurotrophic factor (BDNF), a protein with neuroprotective properties.

The neural pathway involves the vagus nerve, the ENS, and neurotransmitter activity in the GI tract [16]. The vagus nerve has been considered a crucial neural pathway responsible for the bidirectional communication between the gut and brain and between the gut microbiome and the brain [34]. The vagal afferent neurons send signals from the gut to the brain, while the vagal efferent cells transmit signals from the brain to the gut. The vagal afferent pathways influence the HPA axis, which is responsible for adaptive stress responses. Both environmental stress and increased levels of systemic pro-inflammatory cytokines trigger the release of corticotropin-releasing factor (CRF) from the hypothalamus, resulting in activation of the HPA axis. Furthermore, CRF triggers the secretion of adrenocorticotrophic hormone from the pituitary gland, leading to the release of cortisol from the adrenal cortex [35]. Neuronal modulation of afferent sensory nerves can result in local production of neurotransmitters in the gut, including GABA, histamine, acetylcholine, serotonin, and melatonin [16].

Finally, the immune system is a mediator in maintaining a dynamic equilibrium between the brain and the gut. Direct interaction has been reported between the immune system and the HPA axis, afferent nervous system, and ENS [34]. Host–microbiota interactions can result in modulation of immune homeostasis, which can alter brain function via the HPA axis [36,37]. The gut microbiome is thought to influence the metabolism of inflammatory mediators, e.g., the release of cytokines (interleukin (IL)-10 and IL-4) and interferon gamma during dysbiosis [16]. Moreover, the gut microbiota maintains the homeostasis of microglia, which are the innate immune cells of the CNS [13,25].

### 1.2. Correlation between Gut Microbiome and Mental Disorders

Subjects with depression, anxiety, and mood disorders show distinct compositional changes in their gut bacteria profile, raising the question about a possible etiological role of the microbiome in these disorders [38]. Differences in the gut microbial community composition have been observed in patients with mental health conditions such as depression and post-traumatic stress disorder and neurodevelopmental conditions such as autism [11,39]. Alterations in gut microbial profiles have been observed in various preclinical models of brain disorders and can, at least partially, be translated to humans. Recent animal studies have shown that fecal microbiota transplants (FMTs) can transfer behavioral types and emotional states. For example, FMT from depressed patients into germ-free mice has been associated with apparent depressive-like symptoms in the receiving animals [40]. Gut microbiota diversity reduction has been linked to a significant decrease in BDNF, vasopressin, and oxytocin expression in the brain, resulting in behavioral changes in adolescent mice [12]. The mechanisms by which an altered gut microbiome acts on brain development and function are summarized in Figure 1b [12,17,19].

Depression is a multifactorial disorder that involves various pathophysiological conditions [27]. Four major hallmarks of the pathophysiology of major depressive disorders (MDDs) are central dopamine levels, inflammation, stress responses via the HPA axis and the autonomic nervous system, and dysfunction of BDNF [41]. MDD is considered, in some sense, to be a chronic inflammatory disease with altered levels of serum cytokines [42,43]. One animal study showed an association between MDD and several inflammatory pathways, including the nuclear factor κβ (NF-κβ), tumor necrosis factor (TNF), and Toll-like receptor pathways [42]. Chronic stress is associated with extensive gut permeability (leaky gut), leading to neural inflammation via Toll-like receptor-4 [41]. Moreover, in a mouse study, the gut microbiota was found to affect BBB permeability by regulating the expression of the tight junction proteins (TJPs) occludin and claudin-5 in the hippocampus, frontal cortex, and striatum. Enhanced BBB permeability allows inflammatory mediators to enter the brain, leading to neural inflammation [41]. On the other hand, depression is associated with reduced levels of neurotransmitters such as serotonin, dopamine, and noradrenaline, with altered tryptophan metabolism and BDNF levels [14,27,41].

### 1.3. The Beneficial Effect of Gut Microbiome Modulation on Mental Disorders

Alterations in behavior have been observed in experimental animals given certain probiotic bacterial strains [44,45,46]. In addition, human studies have shown the potential translatability of these findings [32,47].

MDD patients show considerable alterations in the presence of several bacterial genera within the Bacteroidetes, Firmicutes, Proteobacteria, and Actinobacteria phyla [48]. One study revealed that in mice with stress-induced HPA axis dysfunction, administration of a probiotic *Lactobacillus* strain elevated BDNF levels, leading to improved glucocorticoid regulation of the HPA axis [49]. A study performed in rats and humans showed that the consumption of a probiotic formulation containing *L. helveticus* and *Bifidobacterium longum* led to anxiolytic-like activity in rats and beneficial psychological effects in healthy human volunteers, indicating an association between the gut microbiota and stress, depression, and anxiety [50]. Moreover, a randomized, placebo-controlled trial of a multispecies probiotic in 40 participants found significant changes in mood, such as reduced sad mood and aggressive thoughts [51].

Gut microorganisms are easily accessible and can be modulated in a variety of ways including the use of probiotics, prebiotics, and dietary measures. Evidence is emerging that the gut microbiome may represent a new target for mental homeostasis, and the term “psychobiotic” has been coined to describe bacteria which confer mental health benefits. Psychobiotics have demonstrated the ability to improve mood, reduce anxiety, and enhance cognitive function in both healthy populations and patient groups. While the term psychobiotics originally referred to beneficial live organisms such as bacteria which are specifically beneficial for mental health [52], the definition has been expanded in recent years to include prebiotics whose effect on the brain is bacteria-mediated [38]. Prebiotics are defined as substrates selectively utilized by host microorganisms conferring a health benefit [53], such as non-digestible carbohydrates or plant polyphenols. It is also worthwhile considering a wider definition of psychobiotics to include any substance that exerts a microbiome-mediated psychological effect, or at least possesses psychobiotic properties, such as probiotics, prebiotics, synbiotics, and postbiotics [39,54].

With this in mind, medicinal plants are obvious candidates for potential psychobiotics that could exert beneficial effects on mental health by interacting with gut microbiota and thereby targeting the MGBA.

Medicinal plants contain complex mixtures of constituents. Many of these compounds have low oral bioavailability. Some are only poorly absorbed in the upper intestinal tract because of their comparably high molecular weight and polarity. Others are absorbed but subject to extensive first-pass metabolism, followed by biliary secretion [55]. These compounds come into contact with the colon microbiota, and a two-way interaction can occur. On the one hand, gut bacteria can decompose plant constituents because of their enormous enzymatic capacities, resulting in the generation of metabolites with altered bioavailability and pharmacological activity profiles. On the other hand, plant constituents may affect the composition and function of the gut microbial community, resulting in, for example, increased levels of health-beneficial bacteria of microbiota-related metabolites [56,57].

Therefore, the term phyto-psychobiotics could be used to describe medicinal plants whose mental effects are mediated via gut microbiota modulation by prebiotic-like effects, postbiotic-like effects mediated by the active secondary metabolites produced by the gut microbiome from the non-digestible herbal ingredients, or even by antibiotic-like effects as in the case with some medicinal herbs that have a mental impact by reducing the level of pathogenic bacteria [58,59].

The aim of this review was to assess the available scientific literature for potential links between the efficacy of medicinal plants used for mental health conditions and their interaction with gut microbiota. For this purpose, we scrutinized published data from clinical studies of medicinal plants for mental disorders and from studies assessing the interaction of these plants with gut microbiota.

## 2. Materials and Methods

**Figure 2:**
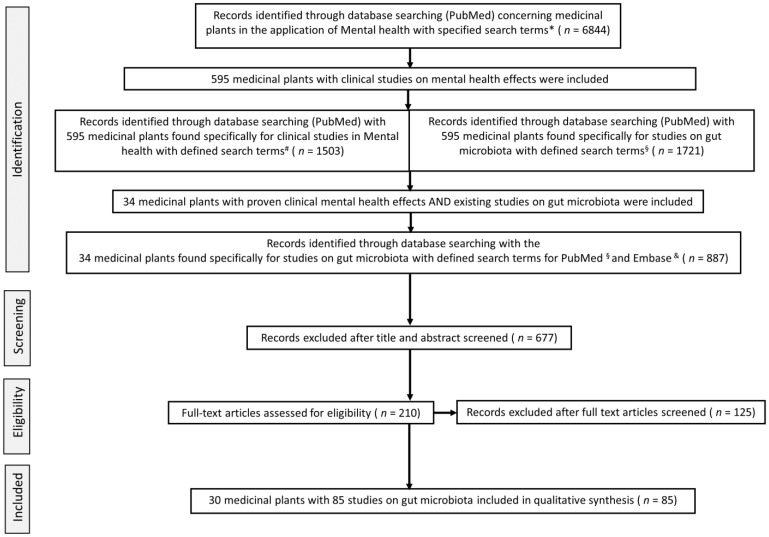
Flowchart of the selection strategy and method (PRISMA statement). * Search terms were as follows: ((medicinal plant *) AND ((antidepressant) OR (mental stress) OR (mood disorder *) OR (insomnia) OR (sleep) OR (anxiety) OR (cognitive impairment *) OR (circadian clock) OR (circadian rhythm) OR (dementia) OR (memory) OR (adaptogen *) OR (focus and attention) OR (fatigue)) NOT ((Alzheimer’s disease *) NOT (Parkinson’s disease *)). ^#^ Search terms were as follows: (plant name OR plant name OR ……) AND (clinical study) AND ((anxiety) OR (insomnia) OR (antidepressant) OR (cognitive impairment *) OR (fatigue) OR (memory)). ^§^ Search terms were as follows: (plant name OR plant name OR ……) AND ((gut microbiome) OR (gut microbiota) OR (gut bacteria)). ^&^ Search terms were as follows: “plant name” AND (“gut microbiome” OR “gut microbiota” OR “gut bacteria” OR “intestinal flora”).

This systematic review is reported according to the Preferred Reporting Items for Systematic Review and Meta-Analyses (PRISMA) statement (Figure 2) to ensure a standardized reporting quality [60].

### 2.1. Eligibility Criteria

Inclusion and exclusion criteria of studies were as follows: Medicinal plants were included in the systematic survey if there was clinical evidence for effects on depression, sleep, anxiety, mood, or cognitive dysfunction and there were studies available (in vitro studies, in vivo studies involving humans and animals except for ruminants and birds) that evaluated an interaction of these medicinal plants and gut microbiota. Only studies performed with the listed plant parts or extracts were considered relevant. No studies on combinations of herbal extracts were included. In the literature survey in Table 1, Table 2 and Table 3 we also excluded published data on pure compounds occurring as main constituents in these extracts but mention them in the discussion of the results when relevant. Studies concerning neurodegenerative diseases such as Alzheimer’s and Parkinson’s were excluded because the neurodegenerative nature of these diseases places them in a separate category.

### 2.2. Search Strategy

Data were successively gathered from the PubMed/Medline and Embase databases (https://www.ncbi.nlm.nih.gov/pmc; https://www.embase.com; last accessed: 5 January 2021). The reference lists of all retrieved review articles were also checked for additional related articles. For the first aim of retrieving all studies dealing with the effects of medicinal plants on mental health, the following search strategy, steps, and general keywords were used in PubMed: ((medicinal plant *) AND ((antidepressant) OR (mental stress) OR (mood disorder *) OR (insomnia) OR (sleep) OR (anxiety) OR (cognitive impairment *) OR (circadian clock) OR (circadian rhythm) OR (dementia) OR (memory) OR (adaptogen *) OR (focus and attention) OR (fatigue)) NOT ((Alzheimer’s disease *) NOT (Parkinson’s disease *)). In the second step, the focus was on clinical effects of the mental health-related medicinal plants identified from the studies retrieved in the first step. Their botanical plant names were specifically searched in PubMed using the following search string: (plant name OR plant name OR ……) AND (clinical study) AND (anxiety) OR (insomnia) OR (antidepressant) OR (cognitive impairment *) OR (fatigue) OR (memory)).

The third goal was the identification of published data on the interaction of the identified medicinal plants and the gut microbiome. The relevant literature was searched in PubMed and in Embase. For PubMed, search terms were (plant name OR plant name OR ……) AND ((gut microbiome) OR (gut microbiota) OR (gut bacteria)); for Embase, search terms were “plant name” AND “gut microbiome” OR “gut microbiota” OR “gut bacteria” OR “intestine flora”.

In the last step, the medicinal plants with reported clinical mental health effects and that were also evaluated in studies of the gut microbiome were selected.. The search strategy is shown in the PRISMA flowchart in Figure 2. The searched data were transferred to the Citavi literature management program.

### 2.3. Study Selection

The titles of all retrieved papers were examined, and studies inconsistent with the objectives of this systematic review were excluded. In the next step, the abstracts of the remaining studies were examined, and again, incompatible studies not meeting the inclusion criteria (see Section 2.1) were excluded. Then, data were extracted from the full texts of the compatible studies and tabulated using standardized information, such as botanical names, medicinal plant parts used, common or local name(s), main constituents, and the field in which the clinical studies had been conducted.

## 3. Results and Discussion

A total of 6844 records were identified from the database searches concerning medicinal plants used for mental health, with 1503 articles related specifically to clinical studies. The second search was for studies of gut microbiota that included the use of these plants with mental health effects, yielding 34 medicinal plants with 887 records. Of these articles, after screening of the title and abstract, 677 were excluded based on the criteria described above (Section 2.1). The remaining 210 full-text articles were further reviewed and screened based on the inclusion criteria, yielding 85 articles on gut microbiome interactions with 30 mental health-related medicinal plants for inclusion in this systematic review. The flowchart of the included studies is depicted in Figure 2.

Table 1 displays the list of the 30 medicinal plants with a clinically proven impact on mental health and for which studies on gut microbiome interactions were available. The included studies on gut microbiota were performed with the same plant parts or extracts as used in the clinical studies. In vitro and in vivo data on gut microbiome interactions are detailed in Table 2 and Table 3.

In vitro studies
  Of the 16 in vitro studies that met the inclusion criteria, 12 were performed with colon microorganisms from human fecal samples. Nine of these twelve studies used single fecal samples from either one or several donors, and the remaining three used pooled fecal samples. In the four nonhuman studies, three used fecal samples from different experimental animals (rat, mouse, dog), and one study applied a set of single microbial strains representing major intestinal genera [88].
  A total of 14 of the 16 studies used simple static batch fermentations, preceded in 4 cases by static simulation of upper GI tract digestion [66,168,191,201]. Another two studies applied more sophisticated dynamic digestion models with sequential upper intestinal tract digestion and colonic fermentation [182,193].
  Nine of the sixteen in vitro studies assessed both the microbial composition and metabolite changes during incubation with a herbal material. Of the remaining seven, three assessed only microbiome changes, and four investigated only metabolite profile changes during incubation.
  The metabolites most often studied in vitro were the SCFAs formed by gut microbial metabolization of plant polysaccharides, followed by metabolites derived from polyphenols and triterpenes.
  Microbial community composition changes were most frequently monitored by 16S rRNA gene sequencing (six studies), fluorescence in situ hybridization (FISH) (four studies), or qPCR (three studies). The study with single strains used cultivation-based agar dilution.
In vivo studies
  Of the 69 in vivo studies that met the inclusion criteria, 11 were clinical, and 58 involved various experimental animal species (34 in mice, 15 in rats, 5 in pigs, and 1 each in rabbits, dogs, *C. elegans*, and *Drosophila*).
  The human studies enrolled comparatively small participant numbers, with intervention group sizes ranging from 6 to 38. Different intervention groups (i.e., placebo vs. treatment or different treatments) were compared in only three of these studies, whereas the remaining eight assessed different treatments in a crossover design or compared the effect of a certain treatment on gut microbiota or metabolite profiles in samples taken before and after the intervention. In all studies, fecal samples were collected for assessment of fecal microbiota changes (seven studies), metabolite changes (two), or both (two). Ten of the studies enrolled healthy (in some cases overweight) patients, and one study enrolled participants with type 2 diabetes mellitus. This latter study assessed the effect of a herbal intervention on depression scores and on the GI tract microbiome composition [68], and thus is the only human study that directly investigated a correlation between a mental health condition and the gut microbial community composition.
  Most of the in vivo studies in experimental animals involved mice and rats. In general, the same bacterial phyla occur in rodents and humans, predominantly Bacteroidetes and Firmicutes. The Clostridium superfamily is also widespread in rats and humans, but there are marked differences in the abundance of important genera such as Lactobacillus and Bifidobacterium between humans and rodents [202,203].
  Of these 58 studies, 27 used healthy animals, and 31 relied on different disease models, most commonly obese animals and colitis induced by dextran sodium sulfate (DSS), along with models of diabetes mellitus type 2, hypercholesterolemia, nonalcoholic fatty liver disease, menopause, and colorectal cancer. In five of the studies, the effects of medicinal plants on the gut microbiota in animal models were assessed related to mental health disorders, such as depression-like behavior, anxiety- and depression-like behavior, and memory impairment [42,106,172,173,204]. Changes in the gut microbial community composition were investigated in 33 of these studies, metabolite changes in 4, and both metabolite and microbial community changes in 21, all with fecal samples from the living animals or fecal content or mucosa from different intestinal regions collected after sacrifice.
  The technique most widely used to assess microbiota changes in human and animal studies was 16S rRNA gene sequencing (applied in 43 studies). Other commonly used techniques were qPCR with primers targeting specific bacterial groups or genera, and cultivation-based methods (bacterial plate counting, agar dilution).
  The microbial metabolites most commonly studied were SCFAs, the microbial fermentation products of polysaccharides (determined in 23 in vivo studies). In some of the studies, microbial metabolites of secondary plant metabolites such as ginsenosides [148,150] or phenolic compounds [200] were investigated.
  In the following sections, we group the data on MGBA interactions of herbal drugs into the major secondary metabolites present in these plants.

### 3.1. Herbal Drugs Rich in Terpenoids

#### 3.1.1. Herbal Drugs Containing Saponins

Many herbal drugs from medicinal plants with clinical effects in mild depression, anxiety, cognitive impairment, insomnia, and fatigue contain triterpenoid saponins (Radix Astragali, Herba Centellae, Radix Ginseng, Radix Polygalae, Folium Gynostemmae) or steroid saponins (Radix Polygonatae, Semen Foenugraeci). Saponins have long been of interest for their potential therapeutic benefits in many diseases, but their poor pharmacokinetic properties, with an extremely low bioavailability (frequently < 1.0%), have hampered the translation of these compounds into drugs. Mechanisms of action of saponin-rich plants on the CNS are largely unknown, and their metabolizaton by and modification of gut microbiota have therefore emerged as potential targets.

*Trigonella foenum-graecum* L. and *Polygonatum sibiricum* Redoutè are medicinal plants with effects on mental health that contain substantial amounts of steroidal saponins. *T. foenum-graecum* substantially corrected the dysbiotic effect of a high-fat diet (HFD) in mice, especially regarding the Firmicutes phylum [177]. The addition of *T. foenum-graecum* to feed positively influenced the gut microbiome composition and immune parameters in weaning piglets [178], and in cultivation-based plate count assays, a saponin-rich *P. sibiricum* extract increased the abundance of probiotic bacteria and decreased the abundance of potentially harmful species [164].

*Polygala tenuifolia* Willdenow is mainly used as a standardized ethanolic root extract (BT-11) that is rich in triterpene saponins and has neuroprotective and antidepressant effects [158,159]. Upon in vitro incubation with intestinal bacteria, 25 triterpene metabolites formed by deglycosylation and deacetylation reactions could be detected [162]. In rats, 29 triterpene metabolites were identified in feces after the administration of an ethanolic *P. tenuifolia* root extract, indicating that these metabolites are not absorbed in vivo but can have local effects on the intestinal microbiome. The altered microbiome may, in turn, indirectly affect brain function through the MGBA [160].

*Astragalus membranaceus* root contains triterpene saponins with the marker compound astragaloside IV, in addition to various compound classes such as flavonoids, polysaccharides, and amino acids.

The authors of one animal study found a significant increase in gut microbiota richness and diversity in a mouse model of type 2 diabetes and a significantly altered relative abundance of several bacterial taxa, inducing an increased abundance of *Lactobacillus* and *Bifidobacterium* [71]. Increases in both genera have been associated with mental health.

Leaf extracts of *Gynostemma pentaphyllum* (Thunb.) Makino, another mental health-related, triterpene saponin-rich medicinal plant, also significantly increased *Bifidobacterium* and *Lactobacillus* abundance and displayed prebiotic-like effects with a significant growth stimulation of SCFA-producing bacteria [120]. Furthermore, in a murine colon cancer model, treatment with *G. pentaphyllum* saponins led to an increase in potentially health-beneficial bacteria, and significantly reduced sulfate-reducing bacteria [116,119]. In addition, treatment with *G. pentaphyllum* saponins increased the Bacteroidetes/Firmicutes ratio in normal [120] and HFD-fed animals [115,118]. Similar to *G. pentaphyllum*, treatment with *P. tenuifolia* root aqueous extract increased the Bacteroidetes/Firmicutes ratio in HFD-fed mice [161]. The aerial parts of *Centella asiatica* (L.) Urban, a herbal brain tonic for mental disorders [80], significantly reduced stress-related depression and anxiety [81]. *C. asiatica* is rich in triterpenoids, specifically asiaticoside, and has shown gut microbiota-modulating properties in a murine colitis model [82].

The best examined medicinal drug influencing the brain and nervous system is ginseng root from Asian ginseng (*Panax ginseng* C.A. Mey.) or American ginseng (*Panax quinquefolius* L.). Numerous randomized, double-blind, placebo-controlled trials have evaluated the efficacy of ginseng for cognitive performance, neurotransmission modulation, memory and learning enhancement, and neuroprotection. Effects have been attributed to a group of ginseng-specific triterpenoid saponins known as ginsenosides. Based on their structures, they are classified into three groups: panaxadiols, panaxatriols, and oleanolic acids [141,146,147].

Ginseng root extracts exert prebiotic-like effects by increasing the abundance of *Lactobacillus* and *Bifidobacterium* in rats [120,142,144] and support the restoration of the intestinal microbiome in antibiotic-treated mice [149]. Recent studies have demonstrated a link between the community structure of the gut microbiome and the gut microbial metabolism of ginsenosides. The three most abundant gut microbial metabolites are ginsenoside Rg_3_, ginsenoside F2, and compound K, formed from the protopanaxadiol group through stepwise cleavage of the sugar moieties [153]. Very high levels of compound K and low levels of the progenitor compound ginsenoside Rb1 were found in human feces after oral administration of American ginseng in healthy volunteers [150].

Host-related factors such as stress or diet lead to changes in the gut microbiome composition and function, which affect the efficiency of ginsenoside metabolism and absorption. Different dietary habits may result in differing gut microbiota populations, in turn affecting gut microbial metabolism and absorption of herbal constituents. For example, distinct fecal levels of ginsenoside Rb1 and compound K have been observed in healthy volunteers with dissimilar dietary habits [151]. After oral administration of an ethanolic extract of American ginseng, compound K was undetectable in antibiotic-treated mice but could be detected in stool samples from vehicle-treated mice [148]. Rats with different degrees of gut microbial metabolism of ginsenosides to compound K have shown different gut microbiome compositions. Isolated colonic *Bifidobacterium* spp. exhibited converting activity of ginsenosides Rb1, Rb2, and Rc to compound K [145]. According to a recent literature review, the main gut microbial genera involved in ginsenoside biotransformation are *Bacteroides*, *Bifidobacterium*, and *Eubacterium* [205].

Ginseng saponins such as ginsenosides Rb1 and Rg1, as well as their partially deglycosylated counterparts ginsenoside Rg_3_ and compound K, have shown antidepressant and anxiolytic effects in various animal models via regulation of neurotransmitters (serotonin, norepinephrine, dopamine, GABA), the HPA axis, the glutamatergic system, BDNF, and intracellular signaling pathways in the CNS. They also reduce the secretion of pro-inflammatory factors (IL-1β, IL-6, TNF-α) and increase the production of anti-inflammatory cytokines (IL-4 and IL-10) [206,207]. The question, which of these mental health-beneficial effects are exerted via direct effects and which are due to indirect mechanisms occurring via the MGBA cannot be answered on the basis of currently existing data. One aspect that deserves particular consideration is that compound K, the major gut microbial metabolite of ginseng saponins, has better bioavailability than its progenitor compounds [207,208].

**Figure 3:**
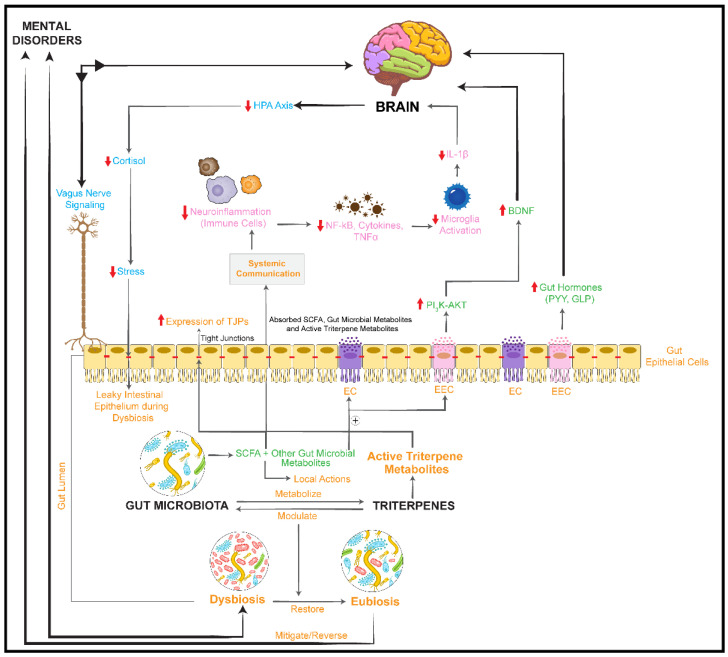
Potential gut–brain communication pathways modulated by triterpenes in mental disorders. Triterpenes (such as ginsenosides) can alter gut–brain microbiome communication in impaired brain function and promote a healthy mental state. These beneficial effects are related to rebalancing the gut microbiome and influencing neural (blue letters), immune (pink letters), and humoral/metabolic (green letters) pathways. Triterpene glycosides are metabolized by the gut microbiome into active components (e.g., ginsenosides into compound K). These active metabolites are more bioavailable than the native compounds. Ginsenosides and their metabolites promote neurotrophic factors and reduce pro-inflammatory mediators and stress levels [207,209,210,211]. The major gut–brain mechanisms by which ginsenosides have a beneficial effect are marked with red arrows (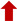 activation/upregulation, 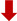 inhibition/downregulation). TJPs: tight junction proteins; BDNF: brain-derived neurotrophic factor; PI3K: phosphoinositol 3 phosphate; AKT: protein kinase B; IL-1β: interleukin-1β; NF-κB: nuclear factor-κB; PYY: peptide YY; GLP1: glucagon-like peptide 1; ⊕: stimulates/promotes.

Taken together, as shown in Figure 3, the available data suggest that triterpenes may modulate an imbalanced microbiome–gut–brain communication during impaired brain functions and promote mental health [207,209,210,211,212,213]. *G. pentaphyllum*, *C. asiatica*, and *P.* *ginseng* exerted prebiotic-like effects and led to a recovered intestinal flora diversity or mitigated gut dysbiosis compared with control groups in rodent models [82,115,116,120,142,144,149].

According to many preclinical studies, certain types of triterpenes possess anti-inflammatory, antioxidant, and antiapoptotic properties and thus may contribute to neuronal protection [206,213]. Moreover, triterpene glycosides can be metabolized by gut microbiota into better absorbable active metabolites that become systemically available [206,207,208].

#### 3.1.2. Essential Oils and Herbs Rich in Essential Oils

Thirteen in vitro and in vivo studies assessing the effect on the gut microbiota of mental health-related essential oils (orange blossom oil, lavender oil) or herbal drugs rich in essential oils (lemon balm leaf, rosemary, lemon verbena leaf, black cumin, and turmeric root) met the inclusion criteria. For lavender (*Lavandula angustifolia* Mill.) oil, traditional use is stated for the indication of sleep disorders, temporary insomnia, mental stress, and mood disorders according to the current European Medicines Agency monograph [214]. Orange, lavender, lemon verbena, and rosemary are used to treat anxiety and insomnia, suggesting anxiolytic and sleep-promoting effects. The most abundant essential oil constituents in bitter orange (*Citrus aurantium* L. ssp. *aurantium*) flowers are limonene and linalool. *D*-Limonene shows antidepressant-like effects by influencing the neuroendocrine, neurotrophic, and monoaminergic systems [215]. In a cultivation-based in vitro study with 12 gut bacterial species, the essential oils of lavender and orange blossom showed preferential inhibitory activity against potentially pathogenic gut microorganisms while having a reduced impact on gut microbes regarded as beneficial [88].

In addition to essential oils, ethyl acetate extracts from *C. aurantium* blossoms contain flavanone glycosides, such as hesperidin, naringin, and neohesperidin. An in vivo study in HFD-fed mice performed with flavonoid-rich extracts indicated a reversal of the HFD-induced gut microbiota imbalance. In particular, the relative abundance of *Bifidobacterium* was increased, and the Firmicutes/Bacteroidetes ratio was significantly decreased [87].

Lemon verbena (*Aloysia citriodora* Paláu) ethanolic extracts contain polyphenols, iridoids, and flavonoids that contribute to their biological effects. In a study with HFD-fed mice, a lemon verbena ethanolic extract reduced intestinal dysbiosis, decreased the Firmicutes/Bacteroidetes ratio, and increased *Akkermansia* abundance in comparison with untreated HFD-fed mice [62]. The biological activities of rosemary (*Salvia rosmarinus* Spenn.) are, on the one hand, related to its volatile constituents and, on the other hand, to phenolic compounds such as the phenolic diterpenes carnosol and carnosic acid, and the phenylpropane derivative rosmarinic acid. Guo et al. (2018) found that supplementation with a rosemary extract containing 60% carnosic acid reduced depression-like behaviors alongside gut microbiota dysbiosis and inflammatory reactions in the hippocampus and serum of chronic restraint stress mice. The microbiome was rebalanced by significantly increasing the abundance of Firmicutes and *Lactobacillus* spp., and by significantly decreasing the abundance of Bacteroidetes and Proteobacteria. The extract exerted an antidepressive effect by suppressing the hippocampal expression of IL-1β, TNF-α, and NF-κB, thus inactivating inflammatory reactions in the hippocampus and microglia. The extract also promoted BDNF and p-AKT/AKT expression in the hippocampus [42].

Two weeks of treatment with an aqueous extract of powdered *Melissa officinalis* yielded an increased microbial Chao-1 diversity index in obese mice. These modifications were associated with higher cecal levels of butyrate, propionate, and ethanol [140].

The rhizome of turmeric (*Curcuma longa* L.) contains volatile oil rich in sesquiterpenes, polysaccharides, and yellow compounds called curcuminoids that have a dicinnamoylmethane skeleton. Petersen et al. studied turmeric powder in an in vitro anaerobic incubation with human fecal microbiota and observed potential prebiotic effects mainly based on the use of the polysaccharides in the herbal material [96]. In an animal study from 1986, colony counts of total aerobes were decreased in rats fed with turmeric, and counts of total anaerobes were increased after 3 months of application [97].

Curcumin has been shown to be metabolized by human fecal bacteria by demethylation, reduction, and hydroxylation reactions [216]. One of these metabolites, di-O-demethylcurcumin, has shown potential neuroprotective effects by attenuating LPS-induced inflammation in rat microglial cells. The metabolite was twofold more active than its parent compound curcumin [217], indicating that curcumin metabolites may have beneficial effects in mental health provided that they are able to pass the BBB.

Many of these findings indicate that it is not the essential oil but rather more polar constituents that are responsible for the interaction with gut microbiota, such as the phenolic diterpene carnosic acid in the case of rosemary, or polysaccharides and curcuminoids in the case of turmeric. This may be because essential oil constituents have a low molecular weight and are rather lipophilic, making them more likely to be absorbed in the upper intestine [218]. Therefore, they are less likely to come into contact with the gut microbiota. Hence, the pronounced mental health-promoting effects of volatile oils [219] may arise via routes other than the MGBA.

#### 3.1.3. Herbal Drugs Containing Other Terpenoids

Extracts of *Ginkgo biloba* L. leaves are used worldwide in a standardized form, containing diterpene lactones (ginkgolides A, B, C, J), the sesquiterpene lactone bilobalide, flavonoids (mainly as glycosides), and polysaccharides. They are applied to neurological disorders connected to impaired cognitive functions and have been considered for anxiety and depression. In an in vitro study with rat intestinal bacteria, the time course of biotransformation of those constituents notably differed among diabetic rats, diabetic nephropathy rats, and healthy rats [107]. The composition and function of gut microbiota can change in response to diseases. If plant constituents are biotransformed by gut microbiota in vivo, their metabolism and absorption in the digestive tract may change with disease-induced changes in the microbial community composition and function. These alterations may, in turn, modulate the systemic effects of these compounds.

To study possible antidepressant mechanisms of *G. biloba*, the efficacy of a polysaccharide fraction from a leaf extract on the gut microbiome composition and depressive symptoms in mice was investigated. Compared with the untreated control group, the extract reduced stress-induced depression and mitigated gut dysbiosis, leading to an enhanced richness of *Lactobacillus*. Oral administration of *L. reuteri* or FMT by oral gavage from ginkgo-treated mice into depressive mice also significantly decreased the immobility time in the forced swimming test. These findings indicate that gut microbiome modulation by *G. biloba* polysaccharides can lead to reduced depressive symptoms, possibly via the MGBA [106].

Saffron (*Crocus sativus* L.) is also used in anxiety, mood disorders, and mild depression, with a considerable number of randomized controlled human clinical trials supporting its application [89,90,91,92,93]. Saffron contains four main bioactive carotenoids: crocin, crocetin, picrocrocin, and safranal, with a lipophilic character that makes them readily absorbable in the upper intestine. Crocin is rapidly hydrolyzed by enzymes in the intestinal epithelium and, to a lesser extent, by gut microbiota, resulting in deglycosylated *trans*-crocetin that is absorbed via the gut mucosa. *trans*-Crocetin is the only saffron metabolite that can cross the BBB and reach the CNS. A pilot study evaluating the effects of saffron on the gut microbiome composition in rats found a strong decrease in the Cyanobacteria and Proteobacteria phyla, and a less dramatic reduction in the Bacteroidetes and Firmicutes phyla [94].

Overall, extracts from *G. biloba* and *C. sativus* mitigated gut dysbiosis and enhanced *Lactobacillus* species compared with untreated control groups in animal studies. The study on *C. sativus* was rather preliminary and performed with healthy rats; the investigation of *G. biloba* was performed with rats that had stress-induced depression behaviors. Because that study was performed with a gingko fraction containing mainly polysaccharides, obviously the polysaccharides and not the terpenes or flavonoids were responsible for the apparent diminution of depressive signs.

### 3.2. Herbal Drugs Rich in Phenolic Constituents

Polyphenols are a broad group of phytochemicals made up of hydroxylated phenyl moieties and present in medicinal plants, tea, fruits, and cereals [27]. The polyphenolic compounds reviewed here belong to three groups: lignans (phenylpropane derivatives), flavonoids (flavan-3-ols, flavanones, flavones, flavone-3-ols, anthocyanidins, and isoflavones), and tannins (derivatives of catechin or gallic acid). Polyphenol esters, glycosides, or polymers are not usually absorbed in the small intestine, and interaction between gut microbiota and dietary polyphenols has often been reported. The gut microbiota can metabolize polyphenols, resulting in the production of potentially active metabolites that can reach the systemic circulation and, in some cases, cross the BBB and exert biological activities [220]. Moreover, polyphenols can alter the gut microbiome composition and function by increasing the population of healthy gut bacteria and decreasing the growth of pathogens, producing a prebiotic-like effect [220].

#### 3.2.1. Herbal Drugs Containing Lignans

Recent clinical trials provide evidence for the use of *Schisandra chinensis* (Turcz.) Baill. and *Eleutherococcus senticosus* (Rupr. & Maxim.) Maxim. in mental (anxiety, depression) and behavioral disorders, including cognitive function, memory, and attention [170]. Most schisandra lignans have a dibenzocyclooctadien skeleton, whereas *E. senticosus* roots contain a mixture of the lignans eleutherosides B4, D, and E, together with phenylpropanoids. In vivo studies indicate that some isolated constituents such as the lignans schisandrin B and eleutheroside E and the phenylpropanoid eleutheroside B contribute to the activity of the total extracts [103].

Studies in rat models have revealed that schisandrin B, the most abundant *S. chinensis* fruit lignan, can cross the BBB thanks to its lipophilic properties and low molecular weight [221]. Apart from lignans, schisandra fruits contain essential oil and polysaccharides. As reported by Yan et al. [173], the total extract and lignans alleviated depressive and anxiety symptoms, whereas the essential oil and polysaccharides ameliorated cognitive decline in lipopolysaccharide (LPS)-induced C57BL/6 mice. These authors also assessed the influence of schisandra total extract, lignans, polysaccharides, and essential oils on the microbiota–gut–brain axis. The total extract (95% ethanol) and the lignan fraction ameliorated depressive-like behaviors by restoring the altered intestinal microbiota composition, enhancing propionate and butyrate concentrations, and exerting anti-inflammatory effects via inhibition of the Toll-like receptor 4/NF-κB/IκB kinase α signaling pathway [173].

Lignans are also the main substances in raw *S. chinensis* fruits and a fruit wine prepared from these fruits, which exerted anxiolytic and antidepressive activities and modulated gut bacterial phylotypes in rats subjected to the chronic unpredictable stress procedure (CUSP). Long-term administration (35 days) restored gut microbial ecosystem dysbiosis occurring in CUSP rats. Of interest, the study authors observed improved cerebral ischemia, enhanced cerebral blood flow, and attenuated hippocampal neuritis after treatment with raw *S. chinensis* fruits and *S. chinensis* fruit wine. Hippocampal neurogenesis is involved in memory and learning, and disrupted neurogenesis is implicated in cognitive impairment and mood disorders, including anxiety and depression [172].

Su et al. also investigated the effect of a *S. chinensis* polysaccharide extract on the composition and diversity of the gut microbiome in mice. The polysaccharides had beneficial effects in mice with ulcerative colitis by recovering the gut microbial profile and increasing SCFA production [175].

In a randomized, double-blind clinical trial with 28 obese women, fecal microbiota community changes after the administration of an aqueous *S. chinensis* fruit extract were found to be different for each participant. This result indicated that *S. chinensis* affected the gut microbiome, but in different ways, depending on the pretreatment gut microbiome composition [174]. Overall, the data suggest that lignans are the most effective fraction of *S. chinensis* in the relief of depressive and anxiety disorders. Their activity may, at least in part, be related to the bidirectional connection between the gut microbiome and the brain. Furthermore, polysaccharide-rich *S. chinensis* extracts were able to reduce the abundance of potentially harmful bacteria through the production of SCFAs and regulate intestinal homeostasis.

#### 3.2.2. Herbal Drugs Containing Flavonoids

Clinical studies indicate that flavonoid consumption may ameliorate mental disorders such as depressive symptoms [222], but the mechanisms involved in these effects have not been fully elucidated. Some flavonoids are orally bioavailable and pass the BBB, and certain flavonoid groups show binding affinity for the benzodiazepine site on the GABA A receptor and inhibit monoaminoxidases A and B [223]. Moreover, flavonoids act as antioxidant agents because of their hydrogen-donating ability, which may ultimately result in neuroprotection [224]. However, a high proportion of flavonoids are not absorbed in the upper intestine and therefore potentially interact with the gut microbiome. These compounds may possess prebiotic effects, since gut bacteria have been reported to be capable of utilizing them [225].

*Glycine max* L. (soy), a medicinal and food plant rich in isoflavones, has shown beneficial effects on mental health in menopausal women [108,109]. An in vivo study in mice showed that feeding with an HFD alone decreased SCFA levels, but this effect was compensated by soy addition. This was accompanied by enhanced relative abundances of Bacteroidetes, which mainly produce acetate and propionate [112]. A study in dogs revealed that soybean husk significantly increased levels of microbial fermentation products such as the SCFAs acetate and butyrate, as well as lactate. In addition, increased abundances of health-beneficial bacteria have been observed in vitro and in vivo [110]. In a rat model of menopause, soy supplementation reduced the Firmicutes/Bacteroidetes ratio and improved cardiometabolic health [113].

Isoflavone glycosides undergo hydrolysis in the upper GI tract and are only partially absorbed. In the colon, unabsorbed isoflavones are decomposed to smaller metabolites, i.e., aglycones and their decomposition products that are formed by reactions such as hydroxylation, hydrogenation, dehydroxylation, and C-ring cleavage [226]. Individual differences in the gut microbiome composition may influence the metabolism of isoflavone aglycones; for example, depending on the gut microbiome composition, daidzein can be further biotransformed either to O-desmethylangolensin or to S-equol, two metabolites with distinct pharmacological activities [227]. Gut microbial isoflavone metabolites may have an impact on mental health. In a placebo-controlled clinical trial in perimenopausal/postmenopausal Japanese women evaluating the effect of pure S-equol supplementation on mood-related menopausal symptoms, the pretreatment anxiety scores of equol producers were lower than those of non-producers, and S-equol supplementation improved mood-related symptoms in equol non-producers [228]. In mice, the microbial daidzein metabolite 6,7,4′-trihydroxyisoflavone improved scopolamine-induced cognitive impairment and enhanced learning memory, possibly by enhancing the expression of BDNF and the phosphorylation of cAMP response element binding, and by reducing acetylcholinesterase and malondialdehyde in the hippocampus [229]. These findings indicate that gut microbial isoflavone metabolites can exert beneficial effects on mental health.

In addition to isoflavones, soybean contains saponins such as soyasaponin I, which has been shown to ameliorate scopolamine-induced memory impairment in mice with intact gut microbiota, although it did not show significant effects in antibiotic-treated animals. Pre-fermentation with the bacterial strain *Lactobacillus pentosus* var. *plantarum* C29 further increased the effect, most likely because the strain can effectively biotransform glycosidic isoflavones and saponins into their more absorbable aglycones [204].

The female inflorescences of *Humulus lupulus* L. (hop) are used as herbal medicinal products for anxiety, mood disorders, and sleep disturbances. Hop contains a mixture of the flavonoids xanthohumol, isoxanthohumol, and 8-prenylnaringenin. These compounds have the potential to modulate and to be metabolized by the gut microbiota [124]. Furthermore, hop extracts comprise primary antimicrobial prenylated phloroglucinol derivatives such as humulones and lupulones. In an in vitro fermentation experiment with a human fecal suspension, a hop extract rich in humulone and lupulone altered the microbial community structure by favoring the growth of Enterobacteriaceae and inhibiting probiotic *Bifidobacteria* and butyrate-producing *Eubacterium*, and reduced butyrate levels. These effects were observed at high hop extract concentrations (final concentration 100–5000 µg/mL), which may be considered nonphysiological [123].

A *Morus alba* L. (mulberry) leaf extract significantly improved working memory and cognitive function in a clinical trial [136]. In an in vivo animal study, changes in the gut microbiome were observed in HFD-induced obese mice. Mulberry leaves partially reversed the microbiome shifts caused by the HFD, significantly increasing the Bacteroidetes/Firmicutes ratio. Additionally, a relative increase in *Akkermansia* and a relative decrease in Proteobacteria were observed [137].

Much of the literature on the interaction between flavonoid-containing plants used for mental health and the gut microbiome focuses on grapes (fruits of *Vitis vinifera* L.). Grape peels and fruit pulp are rich in flavonoids and anthocyanins. Grapes or grape-derived products (e.g., raisins, pomace, extracts) are associated with improved cognitive performance, including attention, language, and memory, as well as calmness and mood [179,180,181]. Several in vitro and in vivo studies showed an influence of grape preparations on the intestinal microbiome, but with different and partly contradictory results.

Mandalari et al. studied in vitro the influence of raisins (dried fruits of *Vitis vinifera*) on the human gut microbiome. Bacterial plate counting showed an increase in *Bifidobacterium* and *Lactobacillus,* and 16S rRNA gene sequencing revealed a relative decrease in Bacteroidetes and *Faecalibacterium prausnitzii*, indicating the potential to promote the proliferation of beneficial bacteria [191]. In contrast, in a human study assessing the effect on the intestinal microbiome of daily raisin consumption for 2 weeks, a significant increase in the relative *F. prausnitzii* abundance was observed, with no consistent relative increase in *Bifidobacterium*. In addition, no significant changes were detected for the Bacteroidetes and Firmicutes phyla in this human study. More pronounced changes were detected after 1 week of raisin consumption rather than after 2 weeks, possibly because raisin ingestion has only short-term effects on the gut microbiome composition [192].

Chacar et al. evaluated the impact of long-term feeding with polyphenol-rich grape pomace extracts on rat intestinal microbiota and observed a potentially more health-beneficial gut microbiome composition in aged rats after 14 months of treatment compared to a control group and young rats [194]. Another study that examined changes in the rat gut microbiome after consumption of polyphenol-rich grape antioxidant dietary fiber (GADF) showed a significant increase in the abundance of *Lactobacillus* spp. [195]. Feeding pigs a diet containing grape seed and grape marc meal extract, a polyphenol-rich byproduct of wine or juice processing, resulted in a reduction in *Streptococcus* abundance and total SCFA levels [196].

Three studies examining the effects of grapes on HFD-induced obesity and gut microbiota in mice showed that ingestion of grape fruit extracts could partially restore the disruption of the intestinal microbiome composition and mitigate many of the adverse health consequences caused by the HFD, such as reduced microbial alpha diversity. Grape administration also influenced the levels of several bacterial families and genera including *Akkermansia*, *Bifidobacterium*, Lachnospiraceae, *Ruminococcus*, and Bacteroidetes [197,198,199]. On the other hand, one study of grape pomace supplementation in healthy women found no changes in the gut microbiome composition. However, a significant increase in SCFAs was observed, likely because of the degradation of fibers or phenolic compounds in the extract. No significant changes were detected in the concentrations of phenolic metabolites, and large inter-individual variations were observed. 3-(4′-Hydroxyphenyl)-propionic acid was the only phenolic compound that clearly increased in the feces of two volunteers after grape pomace supplementation. In the urine, no differences were observed, and plasma samples were not analyzed [200].

The gut microbial metabolites of flavonoids may contribute to the mental health-related activities of medicinal plants. For example, the flavonol metabolites 4-hydroxyphenylacetic acid and 3,4-dihydroxyphenylacetic acid have shown anxiolytic activity in rats after oral and intraperitoneal application, while their progenitor flavonoids kaempferol, myricetin, and quercetin only displayed anxiolytic effects when administered orally, indicating that their gut microbial metabolization is required for activity [230]. The mechanism of anxiolytic action of these metabolites is still unclear, since 3,4-dihydroxyphenylacetic acid has been shown to be unable to cross the intestinal and blood–brain barriers in vitro, and to be rapidly eliminated from plasma in rats [231,232].

In summary, data on the influence of flavonoid-containing, mental health-related medicinal plants on the gut microbiome composition are heterogeneous. Generally, flavonoids are naturally produced by plants to deter bacterial infection and thus likely possess a certain antimicrobial potential towards gut microorganisms. Prenylated hop phloroglucinol derivatives reduced the relative abundances of certain beneficial bacterial genera at high concentrations, whereas isoflavones increased their levels. It is also reported that flavonoids beneficially impact the gut microbial community by increasing the relative abundance of known equol-producing bacteria such as lactobacilli [113]. The highest number of microbiome studies was retrieved for grape extracts and grape products. A large number of intestinal bacterial species were found to be influenced by grape preparations, but the results concerning gut microbiome changes are highly divergent. This may be because of the wide variety of different grape preparations used in the studies and the different experimental platforms for studying the interactions between grapes and the gut microbiome. In summary, studies of the interaction between the gut microbiome and flavonoid-rich grape preparations showed either no significant influence or prebiotic-like effects with no adverse impact on the gut microbiome.

Overall, most studies retrieved on flavonoid-rich, mental health-related medicinal plants were focused on their effects on gut microbiota, while the potential impact of microbial flavonoid metabolites on targets related to the MGBA remained widely unconsidered and deserves a more systematic assessment in the future.

#### 3.2.3. Herbal Drugs Containing Tannins

As already mentioned in Section 3.2.2, grape preparations have positive effects on mental health. While flavonoids and anthocyanins are more abundant in grape peels, grape seeds contain large amounts of condensed tannins.

In an in vitro study with human fecal inoculum, incubation with grape seed polyphenols resulted in a significant increase in potentially beneficial bacteria such as *Bifidobacterium* spp. and *Lactobacillus*-*Enterococcus* groups, while the abundances of *Bacteroides*-*Prevotella* and *Clostridium histolyticum* groups decreased [183]. In contrast, fermentation of grape seed polyphenols in the colonic phase of the GI simulator SHIME, harboring a reproducible human microbial community, led to a general inhibition of the growth of all tested bacterial groups. This inhibition was ascribed to substrate limitation during batch incubation and to a certain antimicrobial capacity that had been previously shown for the applied grape extract [182]. In an in vitro fermentation study, a large proportion of grape seed constituents were found to be indigestible. During in vitro bacterial fermentation with rat cecal inoculum, dietary fibers and proteins were partially degraded, while 97% of the extractable polyphenols were metabolized, leading to the production of SCFAs. Metabolites of the extractable polyphenols were not analyzed in this study [233].

In rats, intake of polymeric grape seed tannins significantly increased the production of SCFAs, whereas the cecal pH and activity of various bacterial enzymes were decreased [184].

Yamakoshi et al. evaluated the effects of a procyanidin-rich grape seed extract on healthy adults after a 2-week administration (0.5 g/day). Culture-based plate counting indicated a significant increase in *Bifidobacterium* and a tendency to decrease for Enterobacteriaceae compared with pretreatment levels [190].

Feeding two doses of grape seed extracts to mice in combination with an HFD showed that grape seed administration could reduce HFD-induced changes in gut microbiota and improve glucose tolerance. Of interest, the lower applied dose seemed to be more effective than the higher one [189]. In ovariectomized mice, administration of a grape seed extract led to an increase in Bacteroidetes and a decrease in Firmicutes, normalizing the Firmicutes/Bacteroidetes ratio [188].

Two studies in pigs investigated the effects of ingesting grape seed meal, the residual from grape seeds after screw pressing the oil. Grosu et al. found that in healthy pigs, the additive increased the relative abundances of Bacteroidetes, Proteobacteria, and *Prevotella* and decreased the relative abundances of Firmicutes, Lachnospiraceae, and *Lactobacillus* [186]. In pigs with DSS-induced colitis, grape seed meal intake attenuated a DSS-induced *Roseburia* increase while stimulating the growth of *Anaerovibrio* and *Megasphaera* and butyric acid production [187].

Choy et al. examined the effects of grape seed extract ingestion on tannin metabolite production and gut microbiota in healthy pigs. The phenolic metabolites detected in feces included hydroxyphenylacetic acids, hydroxyphenylpropionic acids, hydroxyphenylvaleric acids, hydroxybenzoic acids, and caffeic acid. 4-Hydroxyphenylvaleric acid and 3-hydroxybenzoic acid were detected as major phenolic metabolites that increased during grape seed intake compared with baseline [185]. This finding is in line with the results from a study by Sánchez-Pátan et al. with a reproducible human gut microbial community in an in vitro simulator of the human GI tract [182].

Apart from their high levels of lipids, proteins, and dietary fiber, almonds (the seeds of *Amygdalus communis* L.) contain considerable amounts of polyphenols. The most abundant classes are condensed and hydrolyzable tannins (gallotannins, ellagitannins) and flavonoids that are readily metabolized by the human gut microbiota [201]. A randomized controlled trial showed that almonds could ameliorate post-lunch memory decline [63].

An almond-based low-carbohydrate diet significantly improved depression in patients with type 2 diabetes mellitus and induced a significant increase in the growth of SCFA-producing bacterial genera [68]. Psichas et al. reported that SCFAs in combination with free fatty acid receptor 2 can promote the secretion of glucagon-like peptide 1 [234], which is thought to influence depression and anxiety associated with metabolic dysfunction [15]. This finding suggests that the antidepressant effect of almonds may be associated with an increased abundance of SCFA-producing bacteria in the GI tract.

Three other human studies investigated the effect of almond consumption on the gut microbiome, yielding divergent results. Almond snacking for 8 weeks decreased the relative abundance of the opportunistic pathogen *Bacteroides fragilis* in young adults [67]. In another study, the intake of almonds for 18 days led to a decrease in lactic acid bacteria in adults, with no change in the abundance of Bifidobacteria [69]. Holscher et al. reported that the degree of almond processing, such as chopping, roasting, and grinding into butter, differently affected the gut microbiome composition [64].

In vitro fermentation of blanched finely ground almonds and blanched defatted finely ground almonds with human feces led to the conclusion that defatted almonds did not alter the composition of gut microbiota, whereas finely ground almonds stimulated the growth of Bifidobacteria and *Eubacterium rectale* [65]. Similar changes in the gut microbiota with natural and blanched almond skins were found in an in vitro GI digestion and fermentation model with human feces. Almond skins contain polyphenols and high amounts of dietary fiber, with higher polyphenol concentrations in natural than in blanched skins. Therefore, the authors concluded that the dietary fiber present in almond skin rather than polyphenols is responsible for their prebiotic effects [66].

Green tea, prepared from unfermented leaves of *Camellia sinensis* (L.) Kuntze, has a long history of use and is consumed all over the world. Thus, numerous studies have explored the beneficial effects of green tea and green tea extracts, including the modulation of cognitive function and mood in humans, reduced anxiety, improved attention, and cognitive impairment prevention [72,73]. Compounds active in mental health that are found in unfermented green tea leaves are mainly methylxanthines (caffeine), amino acids (*L*-theanine), and flavan-3-ols (main compound: epigallocatechin-3-O-gallate, EGCG). EGCG possesses calming effects and relieves stress, whereas *L*-theanine, especially in combination with caffeine, improves attention and reduces fatigue [72]. High amounts of EGCG and other tea polyphenols are absorbed in the small intestine and undergo metabolism in different organs. The unabsorbed proportion is metabolized by colon microbiota and affects the community composition, inducing potential health-promoting effects due to gut microbiome shifts regarded as beneficial [235].

In four animal studies, changes in the gut microbiome of mice after the administration of green tea leaves or green tea extracts were detected. An aqueous green tea extract partly reversed the HFD-induced changes in the microbial community in mice at the genus and family levels. In addition, it increased total fecal SCFAs, in particular propionic acid and valeric acid [74]. Powdered leaves of purple-leaf tea, a new cultivar of *C. sinensis* with purple leaves, also mitigated the negative effects of an HFD on the murine gut microbiome [75].

In a murine model of chemical-induced colitis, feeding the animals green tea extracts resulted in positive effects on colitis-related signs such as tissue damage and colonic inflammation, and on gut microbiome dysbiosis [76,77]. In addition, the levels of fecal acetic, propionic, and butyric acids were significantly enhanced in one of the studies [76]. In the other study, FMT from green tea-treated to untreated mice also reduced colitis-induced inflammation and tissue damage and mitigated dysbiosis [77].

Additionally, the seeds from *Paullinia cupana* Kunth (guarana) contain tannins and methylxanthines as active compounds. Two double-blind and placebo-controlled studies confirmed the positive effects of standardized guarana seed extracts on mental health due to an improvement in cognitive performance in healthy participants, and on fatigue in breast cancer patients [154,155]. In two animal studies, guarana seed administration was associated with changes in the rat gut microbiome. The findings in one of these studies suggested that ingestion of guarana seed powder for 3 weeks affected the rat gut microbiome in a negative way, increasing the relative abundance of Cyanobacteria and decreasing the relative abundance of *Lactobacillus* and Bacteroidetes, with no impact on microbial diversity. This outcome was attributed to the possible antimicrobial effects of caffeine and other constituents [156]. The authors of the second animal study concluded that guarana administration together with an HFD did not induce considerable changes in the rat gut microbiome [157].

Although this aspect has not been thoroughly investigated in the studies reviewed herein, gut microorganisms are generally known to metabolize flavan-3-ols and condensed tannins from different herbal sources. Therefore, it can be assumed that the flavan-3-ols occurring at high levels in grapes, almonds, and green tea are also degraded by gut microorganisms. Oligo- and polymeric procyanidins are first decomposed to flavan-3-ol monomers, which are degraded by C-ring fission and dehydroxylation steps to dihydroxyphenyl- and hydroxyphenyl-γ-valerolactones and hydroxyphenylvaleric acids. These can be further metabolized to smaller phenolic acids that are also formed during gut microbial metabolism of flavonoids (Section 3.2.2) [236].

To date, there is only a low number of studies assessing the pharmacological effects of phenyl-γ-valerolactones and phenylvaleric acids available in the literature [237]. The study by Unno et al. indicated good BBB permeability for 5-(3,5-dihydroxyphenyl)-γ–valerolactone, the major gut microbial EGCG metabolite in rats. Moreover, this metabolite increased the number of neurites and neurite length in SH-SY5Y neuroblastoma cells, indicating that the compound may promote neurogenesis in the brain [238].

Additionally, hydrolyzable tannins that occur at higher levels in almond skins are known to be metabolized by gut microbiota from studies performed in other tannin-containing plants. Meanwhile, it is well known that ellagitannins are decomposed to ellagic acid and further to urolithins by gut microbiota, with different metabotypes that are capable of producing differing urolithin patterns [239]. Urolithins have been predicted in silico to pass the BBB [240], and they have shown the potential to exert neuroprotective effects mainly in cellular models, but their possible beneficial effects related to mental health still need to be studied systematically [241].

In summary, condensed tannins present in grape seeds can induce changes in the gut microbiome and mitigate gut microbial dysbiosis. However, several studies have shown diverging results regarding changes in the gut microbiome composition, including increased as well as decreased abundances of *Lactobacillus*. Changes in the gut microbiome upon almond intake include an increase in beneficial bacteria such as Bifidobacteria and a decrease in Bacteroidetes, while an antidepressant effect may be related to an increased abundance of SCFA-producing bacteria, since SCFAs stimulate the secretion of the antidepressant glucagone-like-peptide-1. Several animal studies suggest an improvement in microbial dysbiosis and growth promotion of beneficial bacteria by green tea leaves. It remains unclear whether these changes are caused by the methylxanthines or the catechins. Guarana seed intake, on the other hand, did not lead to beneficial effects on the gut microbiome in two animal studies. This may be attributed to the antimicrobial effects of caffeine, but also to the tannins, which possess widely described antimicrobial effects [242]. The role of gut microbial tannin metabolites in mental health-related disorders has not been systematically studied to date.

#### 3.2.4. Herbal Drugs Containing Other Phenolic Compounds

A medicinal herb commonly used to treat depression is *Hypericum perforatum* L. (St. John’s wort). Numerous studies support the role of this plant in the treatment of mild to moderate depression because it has shown comparable efficacy, fewer side effects, and a lower risk of discontinuation when compared with selective serotonin reuptake inhibitors [125]. The plant contains a number of compound classes potentially involved in its antidepressant effects such as hyperforins, polyphenols (including flavonoids such as hyperoside), naphthodianthrones (hypericin), and procyanidins [243]. In a recent animal study, the effects of *H. perforatum* on the gut microbial community composition were investigated in ovariectomized rats. Ingestion of a *H. perforatum* extract could reverse gut microbiome changes at the phylum level caused by ovariectomy-induced estrogen deficiency, and extract application mitigated the increase in the Firmicutes/Bacteroidetes ratio [126].

The roots of *Rhodiola rosea* L. are used as a traditional medicine for their positive mental health effects on anxiety, stress, fatigue, and depression, as shown by in vivo animal and human studies [165,166]. The main phenolic compounds in the roots of *R. rosea* are catechins, procyanidins, and phenylpropanoids (mainly derivatives of cinnamyl alcohols and salidroside) [168]. Labachyan et al. showed that treatment with *R. rosea* root extract could alter the gut microbiome composition in *Drosophila melanogaster* as the order Lactobacillales was significantly decreased and the genus *Acetobacter* was increased [167]. In an in vitro incubation study with human fecal slurry, cinnamylalcohol, tyrosol, and hydroquinone were identified as the main phenolic metabolites [168]. Tyrosol is able to penetrate the BBB and has shown potent neuroprotective and neuroregenerative activities in vitro and in animal studies [244], and hydroquinone has shown protective effects against transient focal cerebral ischemia in rats [245], indicating the neuroprotective potential of these gut microbial *R. rosea* metabolites.

In addition to the well-known administration of *Cannabis sativa* L. for chronic pain and chemotherapy-induced nausea and vomiting, multiple studies have shown an effect on secondary sleep disturbance, although with only moderate evidence [78]. The main active compounds in *C. sativa* are cannabinoids (tetrahydrocannabinol and cannabidiol). Activation of cannabinoid receptors, which are part of the endocannabinoid system, causes multiple changes in GI function including gut motility, gastric secretions, gut–brain signaling, and interactions with the intestinal microbiome, such as increased LPS release [246]. In an animal study, the effects of three cannabis extracts with different cannabinoid concentrations on the gut microbiota composition of mice fed a high-fat/cholesterol diet (HFCD) were examined. The HFCD group receiving a cannabidiol-rich cannabis extract was the only group in which the Bacteroidetes/Firmicutes ratio decreased compared with the control group receiving the HFCD only. The two other extracts, which were either rich in tetrahydrocannabinol or contained similar concentrations of cannabidiol and tetrahydrocannabinol, had no significant impact on the gut microbiome composition [79].

Overall, although numerous studies assessed the interaction of plants with phenolic compounds used for mental health and the gut microbiota, most of them were not designed to assess MGBA-related effects. For only a limited number of plants, such as *Schisandra chinensis* and *Amygdalus communis*, studies are available that indicate potential mediation of mental health-related effects via the MGBA. The impact of the reviewed polyphenol-containing plants on the MGBA is not yet evident from the existing data. However, for many of these reviewed plants, general beneficial and prebiotic-like effects on the gut microbiome have been shown, including mitigation of microbial community imbalances in different animal models of HFD-induced obesity, colitis, and menopause, and the enrichment of potentially health-beneficial bacteria such as SCFA producers, leading to increased intestinal SCFA production. These effects could also be relevant for mental health.

As shown in other studies, the anti-inflammatory activity of polyphenols and the metabolites produced by the gut microbiome can reduce neuroinflammation [27]. Polyphenols and their metabolites can control multiple risk factors for depression (e.g., inflammation, neurotransmitter levels and their precursors, neuronal innervation) and could be beneficial in the prevention and management of different mental health disorders [27,220]. Moreover, in a limited number of studies, gut microbial polyphenol metabolites such as S-Equol, 6,7,4′-trihydroxyisoflavone, 3,4 dihydroxyphenylacetic acid, 4-hydroxyphenylacetic acid, 5-(3,5-dihydroxyphenyl)-γ–valerolactone, hydroquinone, and tyrosol have shown pharmacological effects related to mental health conditions.

**Figure 4:**
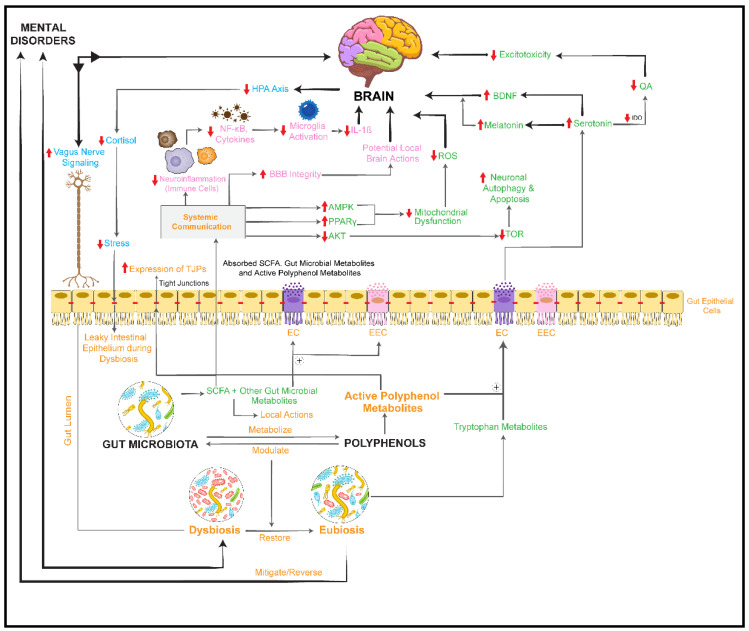
Potential microbiome–gut–brain communication pathways modulated by polyphenols in mental disorders. Gut microorganisms metabolize polyphenols to potentially active metabolites. Polyphenols and their metabolites support the rebalancing of the altered gut microbiome during dysbiosis, and the metabolites can cross the intestinal epithelium and reach the systemic circulation and brain. These molecules may modulate gut–brain communication via neural (blue letters), immune (pink letters), and humoral/metabolic (green letters) pathways. Polyphenols and their metabolites can modulate vagus nerve communication, the HPA axis, pro-inflammatory mediators, neurotrophic factors, and serotonin levels, positively influencing brain functions. Polyphenols have antioxidant effects and can reduce ROS levels in brain disorders [27,220,250], and they can also stimulate gut microbiome production of SCFAs [251]. Furthermore, polyphenols and their metabolites may have local brain effects such as improved cerebrovascular blood flow and a reduction in neuroinflammation [252]. The major gut–brain mechanisms by which polyphenols may exert beneficial effects are indicated with red arrows (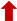 activation/upregulation, 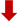 inhibition/downregulation). BBB: blood–brain barrier; IDO: indolamine 2,3 dioxygenase; TDO: tryptophan 2,3-dioxygenase; QA: quinolinic acid; PPARγ: peroxisome proliferator-activated receptor gamma; AMPK: 5′AMP-activated protein kinase; ROS: reactive oxygen species; TOR: target of rapamycin; ⊕: stimulates/promotes.

Figure 4 shows a schematic representation of the key mechanisms of the MGBA through which polyphenols and their microbial-derived metabolites could exert a favorable effect on mental health conditions. Polyphenols exert a prebiotic-like influence on the gut microbiota that may contribute to positive MGBA effects. Moreover, inactive polyphenols are metabolized by gut microbiota to bioavailable and bioactive metabolites [247,248,249]. These active metabolites can reach the systemic circulation by crossing the intestinal epithelium and enhance brain function by regulating pro-inflammatory mediators, the HPA axis, vagus nerve communication, neurotrophic factors, and serotonin levels. Some of them may also permeate the BBB. Moreover, polyphenols may exert antioxidant effects and lower enhanced reactive oxygen species levels in the brain [27,220,250]. In addition, they can stimulate SCFA production by the gut microbiota [251].

### 3.3. Herbal Drugs Rich in Polysaccharides

Dietary fibers are plant polysaccharides that are indigestible in the upper intestinal tract but that can be metabolized by intestinal microorganisms. These fibers and their microbiota-mediated metabolic end products, i.e., SCFAs, can modulate the gut microbiome composition [37]. Traditional medicines rich in polysaccharides that are used to promote mental health include the rhizomes of *Dioscorea opposita* (= *D. oppositifolia* L.; Chinese yam) and the fruits of *Lycium barbarum* L. (goji). A water–ethanol extract from Chinese yam significantly improved conditions such as fatigue, stress, depression, sleep, and calmness [98], while a standardized juice of *L. barbarum* fruits was associated with improved cognitive function, especially semantic fluency [134].

In two animal studies, the administration of Chinese yam significantly restored the disturbance in gut microbiota during or after antibiotic treatment. Zhang et al. assessed the effects of different concentrations of dried Chinese yam powder on antibiotic-treated mice. Ampicillin-induced dysbiosis was restored by ingestion of Chinese yam powder. A significant increase was observed in *Bifidobacteria* and *Lactobacilli*, as was a decrease in *Enterococcus* in the group receiving the highest concentration of Chinese yam [99]. Supplying rats with a Chinese yam water extract together with imipenem/cilastatin sodium increased the abundance of Lachnospiraceae, Ruminococcaceae, Clostridiales, and Firmicutes and decreased the abundance of *Blautia*, *Prevotella*, and *Eisenbergiella* compared with rats receiving only antibiotics [100]. These data indicate the good prebiotic effects of Chinese yam.

Kang et al. showed that goji berry ingestion was associated with considerable changes in the gut microbiota of IL-10-deficient mice, increasing the abundance of butyrate-producing bacteria. Furthermore, the growth of *Bifidobacterium* and the Firmicutes/Bacteroidetes ratio increased. Thus, goji berry demonstrated strong prebiotic effects [135].

**Figure 5:**
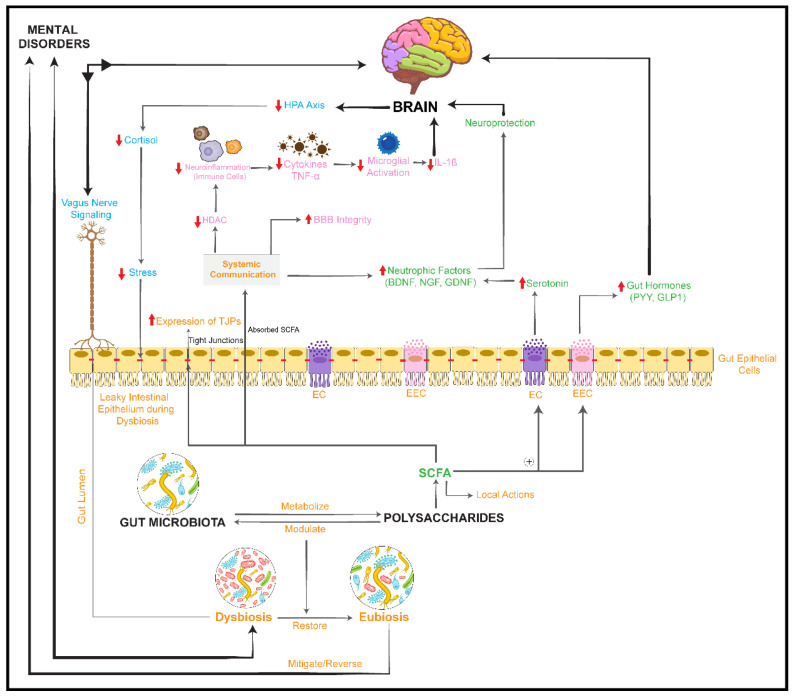
Potential microbiome–gut–brain communication pathways modulated by plant-derived polysaccharides in mental disorders. Gut microorganisms metabolize polysaccharides that resist digestion in the upper gastrointestinal tract into SCFAs. SCFAs modulate gut–brain communication via neural (blue letters), immune (pink letters), and humoral/metabolic (green letters) pathways. SCFAs may reduce cortisol levels, inflammatory mediators, and microglial activation, have a beneficial effect on serotonin synthesis, neurotrophic factors, and various gut neuropeptides, and restore tight junctions in the leaky intestinal epithelium by increasing the expression of tight junction proteins (TJPs). In addition, SCFAs exert local beneficial actions that improve gut health (e.g., maintaining mucus production, anti-inflammatory effects) [37,253,254,255,256]. The major gut–brain mechanisms by which SCFA/active polysaccharide metabolites offer benefit are marked with red arrows (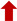 activation/upregulation, 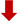 inhibition/downregulation). HDAC: histone deacetylases; GDNF: glial cell-derived neurotrophic factor; NGF: nerve growth factor. ⊕: stimulates/promotes.

As known from other herbal materials, plant-derived polysaccharides that are indigestible in the upper intestinal tract are metabolized by gut microbiota into SCFAs that can influence the gut–brain axis via three major pathways [36,253]. Via the neural pathway, SCFAs can reduce cortisol levels; via the immune pathway, they decrease the levels of inflammatory mediators and microglial activation; and via the humoral/metabolic pathway, they can exert beneficial effects on serotonin synthesis, neurotrophic factors, and various gut neuropeptides. Moreover, SCFAs may restore tight junctions in the leaky intestinal epithelium by increasing the expression of TJPs, and they can exert local beneficial actions on gut health, such as maintaining mucus protection [36,37,253,254,255,256]. A detailed schematic representation of the various pathways describing the possible action of plant polysaccharides (dietary fibers) on the MGBA is presented in Figure 5.

## 4. Conclusions and Outlook

The MGBA is considered a significant therapeutic target for several mental disorders. Medicinal plants contain various classes of secondary plant metabolites, and many of them are poorly absorbed in the upper GI tract due their high polarity and molecular weight. Therefore, most likely, they interact with the gut microbiome and thereby potentially modulate the MGBA. In the present review, 30 medicinal plants showing effects on mental health-related disorders in clinical and animal studies were identified in reports that also showed their potential interaction with the gut microbiota. Overall, 85 in vitro and in vivo studies on this interaction were retrieved.

With a few exceptions, the studies were not designed to directly assess the impact of the respective herbal preparations on targets or pathways related to the MGBA. Nevertheless, they provide indications of a possible interaction with the MGBA, such as positively influencing dysbiotic microbiome conditions, increasing the abundance of health-beneficial or SCFA-producing bacterial species, or exerting anti-inflammatory effects, as in the case of *Salvia rosmarinus*, or because they are metabolized by gut microbiota into active metabolites that affect various MGBA-related pathways, as in the case of ginsenosides.

In some studies, the results indicate that the marker compounds commonly used for their standardization are not responsible for the interaction with the gut microbiome and that other compound classes are involved. For example, in the case of *Ginkgo biloba*, a polysaccharide but not the terpenes or flavonoids obviously exerted positive effects on depressive symptoms in a mouse model of unpredictable chronic mild stress, possibly via modulation of the gut microbiome.

The results of this review indicate that the two-way interaction between the gut microbiome and medicinal herbs could play a role in mediating their mental health effects. We propose that the plant constituents present in these herbs exert their neuroprotective effects through a multitarget effect on the host and the microbiome and can therefore be referred to as phyto-psychobiotics. Certain compound classes such as polyphenols and polysaccharides have been shown to have prebiotic effects. Terms such as flavobiotics and phytobiotics have been used to refer to phytochemical constituents conferring health benefits on the host by positively influencing the gut microbiome [257,258]. Furthermore, recently, it has been proposed that polyphenols act as duplibiotics, meaning that these phytoconstituents have a dual effect on the microbiome by exerting antimicrobial properties, similar to antibiotics, on one hand, and by acting as prebiotics, positively stimulating the growth of beneficial bacteria, on the other hand [259]. Moreover, some of the plant constituents can be metabolized by gut microbiota into pharmacologically active compounds and other postbiotics such as SCFAs, lactate, and phenolic metabolites that can either have a local effect in the gut or be absorbed by the epithelial cells and provide other health benefits to the host via different pathways including the MGBA. Many single plant constituents have been tested for their neuroprotective effects in in vitro and in vivo studies (reviewed elsewhere) [260,261]; however, studies directly assessing the synergistic effects of multiple phytochemical constituents in medicinal plants on MGBA-related targets or pathways are scarce or even non-existent for many candidate plants with clinically proven effects on mental health. Such studies are urgently needed to generate a better understanding of the possible effects of these plants on the MGBA. We recommend that future clinical studies assessing the effect of medicinal plants on mental health should include the analysis of the gut microbiome composition and function to explore the possible action of these medicinal plants on the MBGA. This would facilitate a better understanding of why some individuals respond to interventions while others might be non-responders as they may lack the microorganisms needed to help them metabolize specific plant constituents into active metabolites. Furthermore, combining in vitro GI models, which include both upper and lower GI tract simulation, with multi-omics approaches (e.g., metagenomics, metabolomics, metatranscriptomics, and metaproteomics) can be used as a first step to explore the complex bidirectional interaction between plant constituents and the gut microbiome. These approaches will provide insight into the mode of action and health benefits of herbal medicines, and they will support the identification of new active plant constituents and how they might act via the MGBA or confer additional health benefits on the host.

## Figures and Tables

**Table 1:** Randomized controlled trials and studies of herb–gut microbiome interactions of medicinal plants used in neuropsychiatric disorders.

Botanical Name(s)	Plant Part(s) or Preparation	Common (Local) Name(s)	Dominant Constituent Classes	Application Field in Clinical Studies	Clinical Studies/Reviews	Microbiome Studies
*Aloysia citrodora* Paláu (syn. *Aloysia triphylla* (L’Hér.) Kuntze; *Verbena triphylla* L’Hér.; *Lippia citriodora* Kunth)	folium	lemon verbena leaf	essential oil, phenolic constituents, iridoids, flavonoids	insomnia	[61]	[62]
*Amygdalus communis* L. (syn. *Prunus communis* (L.) Arcang.)	semen	almond	lipids, proteins, dietary fiber, polyphenols	cognitive function	[63]	[64,65,66,67,68,69]
*Astragalus membranaceus* (Fisch.) Bunge *var. mongholicus* (Bge.) Hsiao	radix	membranous milk-vetch root; Huangqi	triterpene saponins, polysaccharides, flavonoids	fatigue	[70]	[71]
*Camellia sinensis* (L.) Kuntze	folium	green tea	methylxanthines, flavonoids, amino acids (theanine)	cognitive function/mood disorders	[72,73]	[74,75,76,77]
*Cannabis sativa* L.	herba	hemp	cannabinoids	insomnia	[78]	[79]
*Centella asiatica* (L.) Urban (syn. *Hydrocotyle asiatica* L.)	herba	Asiatic pennywort, gotu kola	triterpene saponins	anxiety/mood disorders/cognitive function	[80,81]	[82,83]
*Citrus aurantium* L. ssp. *aurantium* (syn. *Citrus aurantium* L. ssp. *amara* Engl.)	aetheroleum (neroli oil)/flos	bitter orange; orange blossom, Seville orange	essential oil, flavonoids	anxiety	[84,85,86]	[87,88]
*Crocus sativus* L.	stigma	saffron	carotenoids (crocines)	depression/anxiety	[89,90,91,92,93]	[94]
*Curcuma longa* L. (syn. *Curcuma domestica* Valeton)	rhizoma	turmeric, curcuma, Indian saffron	curcuminoids, essential oil	cognitive function	[95]	[96,97]
*Dioscorea oppositifolia* L. (syn. *Dioscorea opposita* Thunb.)	rhizoma	Chinese yam	steroid saponins, polysaccharides	cognitive function	[98]	[99,100]
*Eleutherococcus senticosus* (Rupr. et Maxim.) Maxim. (syn. *Acanthopanax senticosus*)	radix et rhizoma	Eleuthero-coccus (Siberian ginseng)	phenylpropanoids, lignans, triterpene saponins, polysaccharides	fatigue and weakness	[101,102,103]	[104]
*Ginkgo biloba* L.	folium	ginkgo leaf	triterpene lactones, flavonoids	anxiety	[105]	[106,107]
*Glycine max* (L.) Merr.	fructus/hypocotyl (soya bean germ)	soya bean; soya flour; soya testa	isoflavones, saponins, proteins, carbohydrates, lipids	depression/insomnia/anxiety	[108,109]	[110,111,112,113]
*Gynostemma pentaphyllum* (Thunb.) Makino	folium		triterpenoid saponins, sterols, flavonoids	anxiety	[114]	[115,116,117,118,119,120,121]
*Humulus lupulus* L.	flos	hop strobile	flavonoids, phloroglucinol derivatives, essential oil	depression/stress/anxiety	[122]	[123,124]
*Hypericum perforatum* L.	herba	St. John’s wort	phloroglucinol derivatives (hyperforin), naphthodianthrones (hypericin), flavonoids	depression	[125]	[126]
*Lavandula angustifolia* Mill. *(L. officinalis* Chaix)	aetheroleum	lavender oil	essential oil	insomnia/anxiety/depression	[127,128,129,130,131,132,133]	[88]
*Lycium barbarum* L.	fructus/fruit juice	GoChi; wolfberry; gouqi; goji berry	polysaccharides, flavonoids, carotenoids	fatigue and weakness/insomnia/stress/depression	[134]	[135]
*Morus alba* L.	folium	mulberry; sang shu	flavonoids	cognitive function	[136]	[137]
*Melissa officinalis* L.	folium	Melissa leaf; lemon balm	essential oil, flavonoids, phenylpropanoids, triterpenes	insomnia/anxiety/mood disorders/cognitive function	[138,139]	[140]
*Panax ginseng* C. A. Meyer.	radix	Korean ginseng; red ginseng	triterpene saponins (ginsenosides), polysaccharides, polyacetylenes	cognitive function	[141]	[120,142,143,144,145]
*Panax quinquefolius* L.	radix	American ginseng	triterpene saponins (ginsenosides)	cognitive function	[146,147]	[148,149,150,151,152,153]
*Paullinia cupana* Kunth ex H.B.K. var *sorbilis* (Mart.) Ducke (=*P. sorbilis* C. Mart.)	semen	guarana seed	methylxanthines, tannins, fatty oil	fatigue/cognitive function	[154,155]	[156,157]
*Polygala tenuifolia* Willdenow	radix	Yuan Zhi	triterpene saponins, phenolic glycosides, xanthones	cognitive function	[158,159]	[160,161,162]
*Polygonatum sibiricum* Redoutè	radix		steroidal saponins, polysaccharides	insomnia	[163]	[164]
*Rhodiola rosea* L. (syn. *Sedum roseum* (L.) Scop.)	rhizoma et radix	arctic root; roseroot; golden root	phenolic glycosides, essential oil, flavonoids	anxiety/stress/cognitive function/depression	[165,166]	[167,168]
*Salvia rosmarinus* Schleid. (syn. *Rosmarinus officinalis* L.)	folium/aetheroleum	rosemary	essential oil, rosmarinic acid derivatives	cognitive function/anxiety/depression/insomnia	[169]	[42]
*Schisandra chinensis* Turcz. (Baill.)	fructus et semen	Wu Wei Zi	lignans, essential oil, polysaccharides	fatigue and weakness	[103,170,171]	[172,173,174,175]
*Trigonella foenum-graecum* L.	semen	fenugreek	polysaccharides, alkaloids, saponins, flavonoids	anxiety	[176]	[177,178]
*Vitis vinifera* L.	fructus et semen	grape seeds; grapes	polyphenols (flavonoids, tannins, stilbenoids)	mood disorders/cognitive function	[179,180,181]	[182,183,184,185,186,187,188,189,190,191,192,193,194,195,196,197,198,199,200]

**Table 2:** In vitro studies of the herb–gut microbiome interactions of medicinal plants used for mental health.

Investigated Plant, Plant Part	Extract, Sample Preparation for Incubation	Preparation of Fecal Samples	Incubation Conditions	Method for Microbiome Analysis	Microbiome Changes	Method for Metabolite Detection	Metabolites	Reference
*Amygdalus communis*, semen	blanched finely ground almonds (FG); blanched defatted finely ground almonds (DG)	fecal material from one healthy donor	fecal batch culture after gastric and duodenal digestion (37 °C, pH 6.8, anaerobic; samples were collected over 24 h)	fluorescent in situ hybridization (FISH) with 16S rRNA-targeted probes for *Bifidobacterium*, *Bacteroides*, *Lactobacillus*/*Enterococcus* spp., *Clostridium histolyticum* group, *Clostridium coccoides*-*Eubacterium rectale* group	increase in *Bifidobacterium* and *E. rectale* in FG group; no change in bacterial composition in DG group	SCFA analysis by HPLC with refractive index detector	increase in lactic acid, butyric acid, acetic acid, and propionic acid in FG and DG groups	[65]
	natural almond skins (NS), blanched almond skins (BS)	fecal material from one healthy donor	fecal batch culture after gastric and duodenal digestion (37 °C, pH 6.8, anaerobic; samples were collected at 0, 4, 8, and 24 h)	FISH with 16S rRNA-targeted probes for *Bifidobacterium*, *Bacteroides*, *Lactobacillus*/*Enterococcus* spp., *Clostridium histolyticum* group, *Clostridium coccoides*-*Eubacterium rectale* group	increase in *Lactobacillus*/*Enterococcus* spp. group, *C. coccoides*-*E. rectale* group, and *Bifidobacteria* in NS and BS group; decrease in *C. histolyticum* group in NS and BS groups	SCFA analysis by HPLC with refractive index detector	increase in total SCFA, lactic acid, acetic acid, propionic acid, and butyric acid in NS and BS groups	[66]
*Centella asiatica*, herba	powdered herb	one pooled sample from twelve healthy vegetarian or vegan women and men; 1% herb or 1% glucose	conditions: anaerobic, 37 °C; pH: 7.4	V3–V4 region of 16S rRNA gene NGS (Illumina); genomic reconstruction of sugar utilization and SCFA pathways	rel. increase: *Enterobacteriaceae* and *Pseudomonadaceae*			[83]
*Citrus aurantium* ssp. *aurantium*, aetheroleum	essential oil	twofold dilutions of essential oil (from 2.0% to 0.004% [*v*/*v*])	conditions: 12 bacterial species representing major intestinal genera on selective agars; 24–72 h cultures	agar dilution method	weak antimicrobial effects on *Bacteroides fragilis*, *Clostridium perfringens*; no antimicrobial effects on *Bifidobacterium*, *Lactobacillus*	-	-	[88]
*Curcuma longa*, rhizoma	powdered rhizome	one pooled sample from twelve healthy vegetarian or vegan women and men; 1% herb	conditions: anaerobic	V3–V4 region of 16S rRNA gene, NGS (Illumina); genome reconstruction of sugar utilization and SCFA pathways	rel. increase at family level: *Bacteroidaceae*, *Desulfovibrionaceae*, *Rikenellaceae*, and *Lachnospiraceae* rel. increase at genus level: *Clostridium* spp., *Bacteroides* spp., *Blautia*, and *Enterobacter* spp. rel. increase in propionate- and butyrate-producing taxa rel. decrease in *Citrobacter freundii*, *Enterococcus faecalis*, *Shigella dysenteriae*, and *Escherichia coli*			[96]
*Ginkgo biloba*, folium	extract with ginkgolides, bilobalide, flavonoid glycosides and aglycones (28.1–0.11 µg/mg)	12 g fresh feces from normal, diabetic, and diabetic nephropathy male Sprague Dawley rats (*n* = 45)	conditions: anaerobic; 37 °C; reaction mixture taken out at 0.5, 1, 2, 4, 6, 8, 12, 16, 22, 28, 36, and 48 h	-	*-*	HPLC-MS/MS	all compounds were biotransformed by rat intestinal bacteria; notably different time course of all 14 compounds in feces of diseased compared to normal rats	[107]
*Glycine max*, fructus	soybean husk; 0.9 mg/g total flavonoids	feces from toy poodle dogs (6.5 ± 3.5 months in age, 2.9 ± 0.4 kg in body weight) (*n* = 3)	conditions: intact soybean husk and enzyme-treated soybean husk; incubated at 39 °C for 24 h	DNA extraction from in vitro cultures; qPCR assay using specific primers	increase: bifidobacteria no effect on total bacteria, total lactobacilli, and *E. coli*	GC-MS for SCFA analysis and D/L-lactic acid assay kit	increase: total SCFAs, including acetate, propionate, and butyrate acids (*p* < 0.01) decrease: indole and skatole acids (*p* < 0.01) no effect on ammonia production	[110]
*Humulus lupulus*, strobile	supercritical CO_2_ extract mixed with canola oil (extract/oil 2:1); hop bitter acids (α-acids/β-acids 1.73:1); tested range 1.5 mg–750 mg hop extract	mixed inoculum from 10 healthy volunteers	conditions: anaerobic, pH: 6.8; sampling after 2.5, 5, 10, 16, and 24 h	qPCR analyses of total bacteria and key bacterial groups; V3–V4 region of 16S rRNA gene NGS (Illumina)	increase: Proteobacteria, Enterobacteriaceae, *Escherichia*/*Shigella*, *Enterobacter*, *Citrobacter*, *Klebsiella* decrease: Lachnospiraceae, Bacteroidetes, *Bacteroides*, Actinobacteria, Firmicutes, *Collinsella*, *Clostridium*, *Eubacterium*, *Desulfovibrio*, *Bifidobacterium*, *Lactobacillus*, *Blautia*, *Dorea*, *Veillonella*, Coriobacteriaceae; *Bacteroides-Prevotella-Porphyromonas* group	analyses of SCFA and other organic acids using HPLC/UV-detection	decrease: total organic acids; butyrate clearly decreased at higher hop concentrations	[123]
*Lavandula angustifolia*, aetheroleum	essential oil	twofold dilutions of essential oil (from 2.0% to 0.004% [*v*/*v*])	conditions: 12 bacterial species representing major intestinal genera on selective agars; 24–72 h cultures	agar dilution method	antimicrobial effects (*Bacteroides fragilis*, *Candida albicans*, *Clostridium perfringens*); no impact on beneficial species	-	-	[88]
*Panax quinquefolius*, radix	ethanolic extract (70%)	6 fecal samples from healthy adult volunteers	conditions: anaerobic, 37 °C; sampling after 24 h incubation	-	-	HPLC/Q-TOF-MS	ginsenoside Rb1 metabolized to compound K and ginsenoside Rg_3_	[149]
	ethanolic extract (70%)	one fresh fecal sample from a healthy Chinese man (28 years old)	conditions: anaerobic, 37 °C; sampling after 24 h incubation	-	-	HPLC/Q-TOF-MS	25 identified metabolites: 13 metabolites were undoubtedly assigned, 12 were tentatively assigned; the 3 most abundant metabolites: 20*S*-ginsenoside Rg_3_, ginsenoside F_2_, and compound K; main metabolic pathways: deglycosylation (stepwise cleavage of sugar moieties), dehydration	[153]
*Polygala tenuifolia*, radix	ethanolic extract (75%)	rat intestinal bacteria with Radix Polygala extract (final concentration of 0.02 g/mL), control, and blank samples	conditions: anaerobic; 37 °C; sampling after 0, 2, 8, 24, 48, 72, or 96 h	V4 region of bacterial 16S rRNA gene, NGS (Illumina); 3 replicates of PCR reactions combined	*Bacteroides* rel. increase more than 60%	UHPLC-IT-MS^n^ and UHPLC-Q-TOF MS	44 detected metabolites: 25 triterpene saponin metabolites (formed by deglycosylation, deacetylation); 16 oligosaccharide ester metabolites; 3 xanthone C-glycoside metabolites	[162]
*Rhodiola rosea,* radix	Methanolic extract (70%)	1 g of human feces in 10 mL of brain heart infusion medium	static upper GI tract digestion, followed by incubation of intestinal phase non-dialyzed retentate in fecal slurries of healthy donors (anaerobic, 37 °C, 48 h)			HPLC-DAD	main metabolites: cinnamyl alcohol, tyrosol, hydroquinone	[168]
*Vitis vinifera*, fructus	red grape polyphenol extract (653 mg gallic acid equivalents (GAE)/g)	fecal samples from two healthy females	dynamic simulator of the GI tract (simgi^®^); extract with or without probiotic supplementation (*Lactobacillus plantarum* CLC-17: 2 × 10^10^ CFU/day); five periods: microbiota stabilization (14 days), extract (800 mg) acute feeding (8 days), probiotic implantation (7 days), extract (800 mg) acute-feeding during probiotic supplementation (8 days), washout (8 days)	16S rRNA gene, NGS (Illumina); bacteria plate counting and qPCR of *Lactobacillus* spp.	increase in Enterobacteriaceae by extract feeding; decrease in Enterobacteriaceae after probiotic implantation; no changes in bacterial diversity after probiotic implantation	targeted analysis of phenolic compounds by UHPLC-ESI-MS/MS and of ammonium ions by ammonium test	increase in phenolic metabolites (benzoic acids) after probiotic implantation; no change in ammonium production	[193]
	sun-dried raisins	fecal sample from one healthy volunteer	upper gastrointestinal digestion followed by fecal batch culture fermentation (37 °C, anaerobic, 24 h)	bacteria plate counting; V4 region of 16S rRNA gene, NGS (Illumina)	sequencing: rel. increase in Proteobacteria, Actinobacteria, and *Roseburia* ssp. rel. decrease in Bacteroidetes, Ruminococcus, and *Faecalibacterium prausnitzii*; plate counting: increase in *Bifidobacteria* and *Lactobacilli*	SCFA analysis by HPLC-RID	increase in total SCFAs, lactic acid, acetic acid, propionic acid, and butyric acid	[191]
*Vitis vinifera*, semen	grape seed polyphenol extract (80% ethanol; 23.5 mg GAE/g)	fecal samples from three healthy volunteers (one female, two males, ages 25–30)	conditions: 37 °C, anaerobic; samples were taken at 0, 12, 24, and 36 h	FISH targeting specific regions of 16S rRNA for total bacteria, *Bifidobacterium* spp., *Lactobacillus-Enterococcus* group, *Bacteroides-Prevotella* group, *Clostridium histolyticum* group, *Eubacterium-Clostridium* group, and *Atopobium* cluster	increase in *Bifidobacterium* spp. and *Lactobacillus-Enterococcus* group; decrease in *Bacteroides-Prevotella* and *Clostridium histolyticum*; no change in total bacteria, *Eubacterium-Clostridium* group, and *Atopobium* cluster	SCFA analysis by HPLC	increase in acetic acid, propionic acid, and butyric acid	[183]
	grape seed extract (GSE; 629 mg GAE/g)	in vitro cultured microbiota with a reproducible human microbial community representative of in vivo conditions	in vitro simulator of the gastrointestinal tract SHIME^®^: ascending colon (AC) and descending colon (DC) compartments; conditions: 37 °C, anaerobic, 48 h; samples were taken at 0, 6, 24, and 48 h	qPCR, specific primers for total bacteria, *Lactobacillus*, *Bifidobacterium*, *Bacteroides*, *Prevotella*, Enterobacteriaceae, *Blautia coccoides-Eubacterium rectale* group, *Clostridium leptum*, and *Ruminococcus*	decrease in all analyzed bacterial groups	SCFA and branched-chain fatty acid (BCFA) analysis by GC-FID; phenolic metabolites by UHPLC-ESI-MS/MS	increase in acetic acid, propionic acid, butyric acid, and total SCFAs and BCFAs in AC; no significant change in SCFAs and BCFAs in DC; steady release of phenylacetic and phenylpropionic acids up to 48 h; formation of flavan-3-ol metabolites	[182]

**Table 3:** In vivo studies of herb–gut microbiome interactions of medicinal plants used for mental health performed in experimental animals or human volunteers.

Investigated Plant, Plant Part	Extract, Sample Preparation	Animal or Study Groups (*n* = Number of Analyzed Individuals)	Animal Species, Volunteers	Conditions	Method for Microbiome Analysis	Microbiome Changes	Method for Metabolite Detection	Metabolites	Reference
*Aloysia citrodora*, folium	ethanolic extract (25%) (LCE)	6 groups: control diet (CD); CD + LCE (25 mg/kg); control high-fat diet (HFD); HFD + LCE (1 mg/kg); HFD + LCE (10 mg/kg); HFD + LCE (25 mg/kg) (*n* = 10 mice per group)	male C57BL/6J mice (7–9 weeks old)	treated for 6 weeks; colonic luminal contents collected	V4–V5 region of 16S rRNA gene, NGS (Illumina)	LCE reduced the enhanced *Firmicutes*/*Bacteroidetes* ratio and relative abundance of *Bacilli* in HFD mice; reversed reduced Bacteroidia, Erysipelotrichia, *Cytophaga*, and *Akkermansia* relative abundances in HFD mice	-	-	[62]
*Amygdalus communis*, semen	almonds	2 groups: low-fat diet (LFD) (*n* = 23); almond-based low-carbohydrate diet (a-LCD); 56 g almonds/day (*n* = 22)	patients with type 2 diabetes mellitus (71.98 ± 5.63 years)	treated for 3 months; fecal samples collected	V4–V5 region of 16S rRNA, gene sequencing (Illumina)	a-LCD: rel. decrease in Bacteroidetes and *Bacteroides*; rel. decrease in *Ruminococcus*, *Eubacterium*, and *Roseburia*	-	-	[68]
	whole, dry-roasted almonds	2 groups: almond group (57 g/day) (*n* = 38); cracker group (77.5 g/day of graham crackers) (*n* = 35)	female and male young adults (BMI 18–41 kg/m^2^; 18–19 years)	treated for 8 weeks; fecal samples collected at baseline and after 8 weeks	V4–V5 region of 16S rRNA, gene sequencing (Illumina)	increase in alpha diversity in the almond group compared to the cracker group rel. decrease in *Bacteroides fragilis*	-	-	[67]
	almonds	three groups: almonds, 0 g/day; 42 g/day; 84 g/day; *n* = 18	healthy adults (10 male, 8 female)	3 feeding periods of 18 days separated by a 2-week washout period; fecal sample collection on first and last days of each feeding period	16S rRNA gene, NGS (454 pyrosequencing), targeting universal primers 27F and 533R; qPCR with specific primers for Bifidobacteria, lactic acid bacteria, and Eubacteria	decrease in lactic acid bacteria by almond consumption; no change in Bifidobacteria by almond consumption	-	-	[69]
	natural almonds; roasted almonds; almond butter	5 periods: 0 g/day of almonds (control diet) (*n* = 18); 42 g/day of whole, natural almonds (*n* = 17); 42 g/day of whole, roasted almonds (*n* = 18); 42 g/day of roasted, chopped almonds (*n* = 15); 42 g/day of almond butter (*n* = 18)	female and male volunteers (BMI 29.7 + 4.4 kg/m^2^; 56.7 + 10.2 years)	5 diet periods of 3 weeks, separated by 1-week non-controlled diet breaks; fecal sample collection at the end of each diet treatment period	V4 region of 16S rRNA gene, NGS (Illumina)	rel. decrease in Actinobacteria, *Bifidobacterium*, and *Parabacteroides* by almond consumption; rel. increase in *Lachnospira*, Roseburia, and *Oscillospira* by chopped almond diet; rel. increase in *Lachnospira* by whole, roasted almond diet; increase in *Dialister* by whole, natural almond diet	-	-	[64]
*Astragalus membranaceus*, radix	fine powder (70% astragalan, 10% total saponins)	two groups: control (0.5% CMC-Na buffer), astragalus (1 g/kg bwd) (*n* = 5 per group)	BKS.Cg-Dock7m +/+ Leprdb/Nju mice (5 weeks old)	treated for 15 days, fresh feces collected	V3–V4 region of 16S rRNA gene, NGS (Illumina) microbial function prediction (PICRUst, KEGG, STAMP)	composition analysis: rel. increased (significant): *Oscillibacter*; *LEfSe*: inhibited growth: *Clostridium* cluster XI; increased growth: *Lactobacillus* and *Bifidobacterium*	-	-	[71]
*Camellia sinensis*, folium	water extracts of green tea (GTWE); black tea (BTWE); oolong tea (OTWE)	5 groups: LFD, 9.4% of calories from fat; HFD, 40% of calories from fat; HFD + 1% GTWE; HFD + 1% BTWE; HFD + 1% OTWE (*n* = 12 per group)	male C57BL/6J mice (7 weeks old)	treated for 28 weeks; fecal samples were collected at week 28	V3–V4 region of 16S rRNA gene, NGS (Illumina)	increase in microbial richness in all tea groups; rel. decrease in Rikenellaceae, Desulfovibrionaceae, *Alistipes*, and *Rikenella* in GTWE group; rel. increase in Lachnospiraceae_NK4A136_group, *Acetatifactor*, and Ruminiclostridium_9 in GTWE group	SCFA analysis by GC	increase in total SCFAs, propionic acid, and valeric acid	[74]
	purple-leaf tea leaf powder (PLT)	4 groups: normal diet (ND); HFD; HFD-1% PLT; HFD-3% PLT (*n* = 8 per group)	male C57BL/6J mice (5 weeks old)	treated for 10 weeks, fecal samples were collected	V3–V4 region of 16S rRNA gene, NGS (Illumina)	HFD-PLT groups compared to HFD group: rel. increase in microbial richness; decrease in Firmicutes/Bacteroidetes ratio; rel. increase in Ruminococcaceae	-	-	[75]
	water extracts from: green tea (GTE); black tea (BTE); yellow tea (YTE); oolong tea (OTE); white tea (WTE); dark tea (DTE); hawk tea (HTE)	9 groups: healthy group; DSS group; GTE + DSS group; WTE + DSS group; YTE + DSS group; OTE + DSS group; BTE + DSS group; DTE + DSS group; HTE + DSS group; (*n* = 6 per group)	Kunming female mice (7–8 weeks old)	treated for 14 days; fecal samples were collected	V3–V4 region of 16S rRNA gene, NGS (Illumina)	in GTE group: increase in microbial diversity; rel. decrease in *Bacteroides*, *Oscillibacter*, *Mucispirillum*, *Helicobacter*, and *Brachyspira*; rel. increase in *Bifidobacterium* and Ruminococcaceae_UCG-014	SCFA analysis by HPLC	increase in acetic acid, propionic acid, and butyric acid	[76]
	green tea water extract (GTE); dark tea water extract (DTE)	3 groups of healthy mice: normal group; GTE (5 mg/kg) group; DTE (5 mg/kg) group	female C57BL/6 mice (7–8 weeks old)	treated for 4 weeks; fecal samples were collected after 4 weeks	V3–V4 region of 16S rRNA gene, NGS (Illumina)	bacterial community richness and diversity unchanged in healthy mice; healthy GTE group: rel. increase in *Lactococcus*, *Akkermansia*, *Lactobacillus intestinalis*, *Alistipes*, and *Parabacteroides distasonis*; rel. decrease in *Turicibacter*, *Romboutsia*, *Allobaculum*, *Ileibacterium*, and *Muribaculum*	-	-	[77]
*Cannabis sativa*, herba	inflorescence extracts (99.9% ethanol): cannabidiol (CBD)-rich CN1 extract; tetrahydrocannabinol (THC)-rich CN2 extract; CN6 extract (CBD/THC ca. 1:1)	5 groups: ND; high-fat + 1% cholesterol + 0.5% cholate diet (HFCD); HFCD diet + CN1 (HFCD+CN1); HFCD diet + CN2 (HFCD+CN2); HFCD diet + CN6 (HFCD+CN6) (*n* = 8 per group)	male C57BL/6J mice (7–8 weeks old)	treated for 6 weeks, 5 mg/kg BW of extract administered every 3 days; cecal contents were collected after sacrifice	V3–V4 region of 16S rRNA gene, NGS (Illumina)	rel. decrease in Bacteroidetes and decrease in Bacteroidetes/Firmicutes ratio in HFCD+CN1 group compared to HFCD group; no significant microbiota changes in HFCD+CN2 and HFCD + CN56	-	-	[79]
*Centella asiatica*, herba	ethanolic extract (75%)	6 groups: control, model group (DSS-induced colitis), DSS+5-aminosalicyclic acid, DSS+*C. asiatica* (100, 200, and 400 mg/kg) (*n* = 8 per group)	male Balb/c mice (22–24 g, 8 weeks old)	treated for 7 days, cecum contents collected after sacrifice	V4 region of 16S rRNA gene NGS (Illumina)	DSS+*C. asiatica* (400 mg/kg): rel. increase: Firmicutes; rel. decrease: Proteobacteria, *Helicobacter*, *Jeotgalicoccus*, and *Staphylococcus*	-	-	[82]
*Citrus aurantium* ssp. *aurantium*, flos	ethanolic extract (85%) partitioned to ethyl acetate subextract (EA)	6 groups: control ND; model control HFD; HFD+ low, middle, and high citrus ethyl acetate (LEA (50 mg/kg), MEA (100 mg/kg), HEA (200 mg/kg)); HFD+simvastatin (*n* = 8 mice per group)	male C57BL/6 mice (weighing 16–17 g, 4 weeks old)	treated for 12 weeks; fresh fecal pellets collected	V3–V4 region of 16S rRNA gene, NGS (Illumina)	HEA increased microbiota diversity and richness; decreased Firmicutes/Bacteroidetes ratio; rel. decrease Erysipelotrichaceae and others rel. increase: Bifidobacteria and others	-	-	[87]
*Crocus sativus*, stigma	saffron (not defined)	two groups: control (water), saffron in drinking water (120 mg/day) (*n* = 10 per group)	rats (not defined)	treated for 4 weeks; stool samples collected before and after 4 weeks	16S rRNA gene NGS (Illumina) using universal bacterial primers	strong rel. reduction: Cyanobacteria, Proteobacteria less strong rel. decrease: Bacteroidetes, Firmicutes rel. increase: Spirochaetes, Tenericutes, *Candidatus saccharri*	-	-	[94]
*Curcuma longa*, rhizoma	turmeric powder (2.5% curcumin); alcoholic turmeric extract containing curcumin and turmeric oil fraction	three groups: control diet (CD); CD + 100 mg turmeric powder; CD + 20 mg turmeric extract (*n* = 10 rats per group)	male Wistar albino rats (21 days old; ≈32 g)	five animals of each group killed after 3 months, others after 2 years; cecal contents collected after sacrifice	agar dilution (0.1% peptone for aerobes; sterile mineral solution for anaerobes)	significant decrease after 3-month treatment: total aerobes, Lactobacilli significant increase after 3-month treatment: total anaerobes, *Clostridium perfringens*, and coliforms significant decrease after 2-year treatment: coliforms	-	-	[97]
*Dioscorea oppositifolia*, rhizoma	dried Chinese yam powder (CY)	five groups: normal control (NC) group (water); model control (MC) group (antibiotic-associated diarrhea, AAD); low-dosage (CL) group (AAD + 4.28 g/kg BW CY suspension); medium-dosage (CM) group (AAD + 8.56 g/kg BW CY suspension); high-dosage (CH) group (25.68 g/kg BW CY suspension) (*n* = 10 per group)	male Balb/c mice (7 weeks old)	days 1–5: MC, CL, CM, and CH groups: ampicillin (22.4 g/kg BW, two times per day); days 6–15: water for MC group, CY for CL, CM, and CH groups; fecal samples were collected	bacterial counting, specific agar plates for Bifido-bacteria, lactobacilli, *Enterococcus*, and *Clostridium perfringens*; denatured gradient gel electrophoresis (DGGE) and V3 region 16S rRNA gene sequen-cing of DGGE target bands	increase in Bifidobacteria and Lactobacilli in CH group; decrease in *Enterococcus* in CH group and *Clostridium perfringens* in CL, CM, and CH groups; increase in *Bacteroides* spp. and *Clostridium* spp. in CL, CM, and CH groups	SCFA analysis by GC-FID	increase in total SCFAs in CL, CM, and CH groups	[99]
	Chinese yam extract (hot water) (CY)	three groups: NC; antibiotic group (A; 50 mg/kg BW imipenem/cilastatin Na); CY group (ADR; 50 mg/kg BW imipenem/cilastatin Na + 3.4 g/kg BW CY) (*n* = 6 per group)	SPF-grade male Wistar rats (100 ± 10 g)	treated for 21 days; fecal samples were collected	V3–V4 region of 16S rRNA gene, NGS (Illumina)	ADR group: increase in microbial diversity reduced by antibiotic; rel. increase in Lachnospiraceae, Ruminococcaceae, Clostridiales, and Firmicutes; rel. decrease in *Blautia*, *Prevotella*, and *Eisenbergiella*	metabolic profile analysis by UPLC-Q-TOF/MS	CY administration returned fecal sample metabolite profile to normal	[100]
*Eleutherococcus senticosus*, plant part not specified	ethanolic extract (EE)	four groups: control, EE (30 g/100 kg), *Enterococcus faecium* AL41 (EFAL41), EFAL41 + EE (*n* = 24 rabbits in each group)	post-weaned rabbits (Hyplus breed) (5 weeks old)	treated for 42 days; fecal sampling on day 0/1 (start of experiment), day 21, and day 42; on days 21 and 42, 3 animals per group were sacrificed	agar dilution methods on specified agars for enterococci, EFAL41, coagulase-negative and coagulase-positive staphylococci, *Clostridium difficile*, coliforms, pseudomonads	EE group: reduction in: coagulase-negative staphylococci and Clostridia on day 21	cecal lactic acid and SCFA analysis using GC (days 21 and 42, 3 animals per group were sacrificed)	different concentrations of propionic acid in all experimental groups in comparison to control on day 42	[104]
*Ginkgo biloba*, folium	polysaccharide-rich water extract (GPS)	stage 1–4 groups: control; unpredictable chronic mild stress mice (UCMS); UCMS + GPS (300 mg/kg BW); UCMS + paroxetine (30 mg/kg BW), (*n* = 10 per group); stage 2 fecal microbiota transplant (2 groups): mixed antibiotics, oral gavage of fecal samples from donor mice (UCMS-FMT or GPS-FMT) (*n* = 8 per group) *Lactobacillus**reuteri* treatment (3 groups): control; UCMS; UCMS + oral gavage of *L. reuteri* (*n* = 8 per group)	male SPF BALB/c mice (3–4 weeks old)	treated for 4 weeks, fresh feces collected; behavioral experiment after 30 days of GPS/paroxetin treatment, FMT, or *L. reuteri* treatment	V3–V4 region of 16S rRNA gene, NGS (pyrosequencing)	antidepressant effect in forced swimming test in UCMS-GPS group vs. UCMS group, and in GPS-FMT group vs. UCMS-FMT group; GPS reversed gut dysbiosis induced by UCMS; 113 differential OTUs between UCMS-GPMS and UCMS groups	-	-	[106]
*Glycine max*, fructus	legume powder; isoflavone content in *Glycine soja* (HFG) 788.77 µg/g, in *Glycine max* (HFB) 139.72 µg/g	four groups: control (normal chow; NCD); standard HFD; HFD with 20% HFG; HFD with 20% HFB (*n* = 12 mice per group)	male C57BL/6J mice (7 weeks old, 18–20 g)	treated for 11 weeks; fresh feces collected in the last week in the morning	V3–V4 region of 16S rRNA gene, NGS (Illumina)	reversal of HFD-induced gut microbiota changes in HFB and HFG rel. increase: *Bacteroidetes*, *Proteobacteria*, *Allobaculum*, *Parasutterella*, *Anaerotruncus*, *Helicobacter*, *Alistipes*; rel. decrease: *Verrucomicrobia*, *Akkermansia*	analysis of fecal SCFA content by HPLC/PDA detector	total SCFA and acid concentrations reduced in HFD group, but elevated in HFG- and HFB- supplemented groups; acetic and propionic acids and total SCFAs higher in HFG than in HFB	[112]
	soybean husk with 0.9 mg/g total flavonoids	two groups: cellulose powder (10 g) or soybean husk powder (5.6% of total diet) (*n* = 4 per group)	healthy Shiba dogs (7–48 months in age and 7.5 ± 1.7 kg in body weight)	treated for 7 days; feces collected on morning and evening of days 6 and 7	qPCR assay using specific primers	increase: total lactobacilli, *Clostridium* cluster IV, *Faecalibacterium prausnitzii*, *Clostridium* cluster XIVa, *Bacteroides-Prevotella-Porphyromonas* group; decrease: *Clostridium* cluster XI	analysis of SCFA by GC-MS; D/L-lactic acid assay	increase: total SCFAs, acetic, butyric, and lactic acids (*p* < 0.05) decrease: indole and skatole	[110]
	soy (590 mg/isoflavones kg diet (genistein and daidzein equivalents))	4 groups: OVX + soy; SHM + soy; OVX + soy-free (control); SHM + soy-free (control) (*n* = 10 rats per group)	female rats bred for low-running capacity, either ovariectomized (OVX) or sham-operated (SHM) (27 weeks old)	treated for 28 weeks; cecal digesta samples collected	V3–V4 region of 16S rRNA gene, NGS (Illumina)	OVX + soy and SHM + soy: rel. increase: *Bacteroidetes*, *Prevotella*, *Lachnospiraceae*, *Dorea*, *Phascolarctobacterium*, *rc4-4*, *Sutterella* rel. decrease: *Firmicutes*, *Coprococcus*, *SMB53*, *Clostridiaceae*, *Desulfovibrionaceae*, *Adlercreutzia*, *Bifidobacterium CF231*, *Desulfovibrio*, *Roseburia*, *Treponema*, Peptostreptococcaceae; lower Firmicutes/Bacteroidetes ratio (*p* < 0.001)	-	-	[113]
*Gynostemma pentaphyllum*, folium	*Gynostemma pentaphyllum* saponins (GpS)	3 FMT donor groups: GpS treatment (Apc+GpS 300 mg/kg BW); non-treatment (Apc-GpS); wild-type (WT) control (C57BL/6J mice—GpS, B6 group) 4 FMT groups: control group (no FMT), B6 FMT, Apc-GpS FMT, and Apc+GpS FMT (*n* = 8 per group)	male C57BL/6J (WT) and Apc^Min/+^ (colon cancer model) mice (4–6 weeks)	treated for 8 weeks; at the end of week 4, fresh feces collected every 3 days from FMT donors; FMT groups received transplants every 3rd day for 4 consecutive weeks	enterobacterial repetitive intergenic consensus (ERIC)-PCR and qPCR with taxon-specific 16S rRNA gene primers	Apc/GpS FMT group: significant increase in *Bacteroides*, Bacteroidetes/Firmicutes ratio, beneficial bacteria such as *Bacteroides*, *Bifidobacterium*, *Lactobacillus*, *Clostridium Cluster IV*, and *Faecalibacterium prausnitzii*			[119]
	*Gynostemma pentaphyllum* saponins (GpS); 50 mg/mL in 0.5% carboxymethyl cellulose	four groups: nonxenograft-control, nonxenograft-GpS (*n* = 6 per group); xenograft-control and xenograft-GpS; (750 mg/kg BW; *n* = 7 per group)	athymic nude mice (BALB/c-nu/nu); xenograft performed by injecting 10^6^ R6/GFP-*ras*-transformed cells into the flank (7 to 8 weeks old)	treated for 12 days; animal feces collected from each mouse for two consecutive hours on day 0 (before xenograft), and day 5 and day 10 after GpS treatment	ERIC-PCR; 3 fecal samples randomly picked from each experimental group on day 10 for further 16S rRNA gene NGS (454 pyrosequencing)	GpS induced alteration in microbiota in xenograft, but not in nonxenograft mice; *Clostridium cocleatum* and *Bacteroides acidifaciens* rel. increase by GpS treatment in xenograft and nonxenograft mice	-	-	[117]
	*Gynostemma pentaphyllum* saponins (GpS); 50 mg/mL in 0.5% carboxymethyl cellulose	three groups: WT-control, WT-GpS, Apc^Min/+^-control, Apc^Min/+^-GpS; 500 mg/kg (*n* = 12 mice per group)	heterozygous male Apc^Min/+^ (C57BL/6J-Apc^Min/+^) and female WT C57BL/6J mice (6 weeks of age)	treated for 8 weeks; fecal samples collected from for two consecutive hours before treatment and weekly after treatment	ERIC-PCR; 5 fecal samples randomly picked from each experimental group on week 8 for further 16S rRNA gene NGS (454 pyrosequencing)	GpS rel. increase: *Bacteroides acidifaciens*, *Bifidobacterium pseudolongum*, *Clostridium cocleatum*, *Lactobacillus intestinalis*, *Parabacteroides distasonis*, *Streptococcus thermophilus*, and Bacteroidetes/Firmicutes ratio GpS rel. decrease: *Acinetobacter lwoffii* and sulfate-reducing bacteria	-	-	[116]
	*Gynostemma pentaphyllum* saponins, saponin content 85% (GpS)	2 groups: control group (water), GpS group (500 mg GS/kg BW 1× per day) (*n* = 10 per group)	male C57BL/6 mice (8 weeks old)	treated for 15 days; feces collected for 2 consecutive hours on days 0, 5, 10, and 15 upon treatment	ERIC-PCR; qPCR with primers targeting 16S rRNA gene of specific bacterial groups	GpS group vs. control: increased: Bacteroidetes, Bacteroidetes/Firmicutes ratio, *Bacteroides* spp., *Lactobacillus* spp., *Faecalibacterium prausnitzii* decreased: Firmicutes	-	-	[120]
	*Gynostemma pentaphyllum* (GP) decocted twice with 4 L water (2 g/mL)	6 groups: control, model group (HFD-induced nonalcoholic fatty liver disease, NAFLD), NAFLD + positive control (22.8 mg/kg DLPC), NAFLD + GP, 6 g/kg BW (GPH), NAFLD+ GP, 3 g/kg BW (GPM); NAFLD + GP, 1.5 g/kg BW (GPL) (*n* = 10 per group)	male adult Sprague Dawley rats (180–220 g)	rats fed with chow diet or HFD for 8 weeks; from week 5, treated for 4 weeks; cecum, contents collected after sacrifice	V3–V4 region of 16S rRNA gene; V4 and V9 regions of 18S rRNA gene, NGS (Illumina); PCR of ITS1 and ITS2 regions	GP treatment shifted microbiota composition towards that of healthy control; GP decreased Firmicutes/Bacteroidetes ratio to a value comparable to healthy control; GP rel. increase: *Lactococcus*; GP rel. decrease: pathogenic bacteria, including *Ruminococcus* spp.	-	-	[118]
	100 g *G. pentaphyllum* dry herb boiled in water (1.25 g/mL) (GP)	3 groups: control (chow diet + water), model group (HFD-induced NAFLD + water), GP treatment group (HFD-induced NAFLD + GP; 11.7 g/kg BW (12 mL GP/kg BW)	male C57BL/6J mice (6 weeks old)	feeding with chow diet or HFD for 28 weeks; treatment from week 13 on; 6 animals per group picked for feces collection (once per day on 3 consecutive days)	V3–V4 region of 16S rRNA gene, NGS (Illumina)	GP restored reduced gut microbial diversity and microbial shifts induced by HFD: rel. decrease in the enhanced Firmicutes levels including genera *Eubacterium*, *Blautia*, *Clostridium*, and *Lactobacillus*; rel. increase in the reduced *Parasutterella* levels	-	-	[115]
*Humulus lupulus*, strobile	hop extract suspended in sesame oil; hop extract (HE) (5.1 mg/g 8-prenylnaringenin, 6.3 mg/g xanthohumol), 400 mg/kg BW	5 groups: OVX placebo (sesame seed oil, *n* = 11), OVX plus HE (*n* = 11), OVX plus 17β-estradiol (*n* = 9), SHAM placebo (sesame seed oil, *n* = 10), SHAM plus HE (*n* = 8)	female C57BL/6 retired breeder mice (7 months old); ovariectomized (OVX) or sham-operated (SHAM)	duration: 12 weeks surgery after week 2; treatment started 4–7 days post-surgery; fecal samples from week 10 (SCFAs), cecal contents (microbiota analysis)	V3–V4 region of 16S rRNA gene, NGS (Illumina)	no influence on total bacterial abundances; rel. decrease *Akkermansia muciniphila* in SHAM plus HE group compared to SHAM placebo and OVX plus 17β-estradiol group; no reduction in OVX plus HE group	SCFA analyses using GC-FID	no significant differences in fecal SCFA levels among groups	[124]
*Hypericum perforatum* L., herba	*H. perforatum* extract (8.94% total flavonoids, 0.026% hyperoside, 0.323% hypericin) (HP)	3 groups: OVX group; OVX-HP group (extract 300 mg/kg BW HP); sham group (*n* = 8 per group)	female Sprague Dawley rats (260–300 g, 6–8 weeks old)	treated for 12 weeks; feces were collected for 3 days before the end of the experiment	V3–V4 region of 16S rRNA gene, NGS (Illumina)	HP group: increased Firmicutes/Bacteroidetes ratio; rel. increase Firmicutes and Verrucomicrobia; rel. decrease Bacteroidetes, Elusimicrobia, and Gemmatimonadetes	SCFA analysis by GC-FID	HP group: increased acetic acid, propionic acid, butyric acid, valeric acid, and hexanoic acid	[126]
*Lycium barbarum* L., fructus	goji berry powder	2 groups: standard rodent diet (Con); Con diet + 1% goji (*n* = 7 per group)	male IL-10-deficient mice (6 weeks old)	treated for 10 weeks; fecal samples (colonic contents) were collected at necropsy	V4 region of 16S rRNA gene, NGS (Illumina)	goji group: increase in Firmicutes/Bacteroidetes ratio; rel. increase in Actinobacteria, Bifidobacteriaceae, Lachnospiraceae, Ruminococcaceae, *Bifidobacterium*, *Clostridium* XVIII, *Roseburia* sp., *Clostridium leptum*, and *Faecalibacterium prausnitzii*; rel. decrease in Peptostreptococcaceae	SCFA analysis by GC-FID	increase in butyric acid and isovaleric acid	[135]
*Melissa officinalis*, folium	lemon balm water extract (LB) (2.76 mg rosmarinic acid/100 mg dried raw material)	2 groups: control (water); LB group (LB dissolved in water, 500 mg LB/day/mouse) (*n* = 5 per group)	C57Bl/6J male ob/ob mice (12 weeks old)	treated for two weeks; gut (fecal) microbiome analyzed before and after treatment	V3–V4 region of 16S rRNA gene, NGS (Illumina)	LB group: increase: Chao-1 diversity index and Porphyromonadaceae	metabolomic analysis of cecum content for SCFAs and other metabolites	significantly higher levels of butyrate, propionate, and ethanol; significantly lower level of lactate	[140]
*Morus alba* L., folium	dried and powdered mulberry leaves	three groups: control group, LFD, 10% calories from fat; HFD, 60% calories from fat; mulberry group (M + HFD; HFD plus 20% M) (*n* = 6 per group)	male C57BL/6J mice (15–20 g, 4 weeks old)	8 weeks until weight difference between HFD and LFD is ca. 20%; treated for 13 weeks; feces collected after adaptation, HFD-induced obese model construction, and at the end	V3–V4 region of 16S rRNA gene, NGS (Illumina)	increase in Bacteroidetes/Firmicutes ratio; rel. decrease in Firmicutes and Proteobacteria; rel. increase in Bacteroidetes and *Akkermansia*	-	-	[137]
*Panax ginseng*, radix	red and white Korean ginseng powder (WG, RG)	three groups: control (basal diet), WG group (7.0% *w*/*w* of diet WG), RG group (7.0% *w*/*w* of diet RG) (*n* = 10 per group)	Sprague Dawley male rats	treated for 21 days, postmortem: ileum contents (anterior to the ileocecal valve) collected	qPCR with primers for all bacteria, *Lactobacillus*, *Bifidobacterium*, *Escherichia coli*, *Clostridium* cluster I, *Bacteroides*-*Prevotella*-*Porphyromonas* group	RG and WG groups: significantly higher number of total bacteria (*p* = 0.014) and *Lactobacillus* strains (*p* = 0.018)	-	-	[144]
	freeze-dried granulated *Panax ginseng* extracts g	*Panax ginseng* extract (4 g two times/day), no placebo group (*n* = 10 women)	women aged 40–60 years and body mass index ≥ 25 kg/m^2^	8-week clinical trial, fresh human stools collected on the 1st visit day (week 0) and the last day (week 8)	V1–V3 region of 16S rRNA gene, NGS (454 pyrosequencing)	rel. abundance of *Anaerostipes* decreased after ginseng intake; subgroup analyses with effective (EWG) and ineffective weight loss groups (IWG): increased in EWG: rel. abundance of *Anaerostipes* and *Eubacterium_g5*; increased in IWG: *Lactobacillus*; rel. abundance of *Bifidobacterium*, *Escherichia*, and *Clostridium_g23* in EWG significantly lower than in IWG			[143]
	ethanolic extract (80%) (PGE)	PGE (100 mg total saponins/kg BW) (*n* = 60 rats), no control group	male Sprague Dawley rats (7 weeks old, weight: 220 ± 20 g)	treated for 12 h; colonic content samples collected	V1–V3 region of 16S rRNA gene, NGS (Illumina)	subgroup with low-efficiency metabolism (LEM) and high-efficiency metabolism (HEM): rel. abundance of Alcaligenaceae, Coriobacteriaceae, Bifidobacteriaceae, S24-7, Erysipelotrichaceae, Peptostreptococcaceae, and Campylobacteraceae significantly higher in HEM; Lachnospiraceae, Prevotellaceae, Porphyromonadaceae, Defluviitaleaceae, Lactobacillaceae, and Veillonellaceae significantly lower in HEM	LC-MS/MS (MRM mode, precursor-product ion pairs)	protopanaxadiol-type ginsenosides: selective elimination of the C-20 and C3- terminal sugar moieties to compound K, or of the C-20 sugar chain to ginsenoside Rg_3_; protopanaxatriol-type ginsenosides: C-20 and C-6 sugar moieties hydrolyzed to protopanaxatriol	[145]
	ginseng extract (not defined)	2 groups: control (distilled water), ginseng extract (100 mg/kg; *n* = 9 per group)	male Wistar rats (34 weeks with 300 g)	treated for 34 weeks, intestinal (cecum, ileum) contents collected after sacrifice	V3 region of 16S rRNA gene, NGS (pyrosequencing with the GS FLX platform)	rel. increase in ginseng group*: Bifidobacterium*, *Lactobacillus*, *Methylobacteriaceae*, and *Parasutterella*	untargeted GC-TOFMS	ginseng group: 25 significantly changed metabolites from cecum and 35 from ileum; upregulated: amino acids, arachidonic acid, polyamines, and organic acids; downregulated: linoelaidic acid, palmtelaidic acid, oleic acid, and glycerol	[142]
	ginseng saponin extract (80% saponins) (GS); red ginseng saponin extract (80% saponins (RGS))	3 groups: control group (water); GS group (500 mg GS/kg BW 1× per day); RGS group (500 mg RGS/kg BW 1× per day) (*n* = 10 per group)	male C57BL/6 mice (8 weeks old)	treated for 15 days; feces collected for 2 consecutive hours on days 0, 5, 10, and 15 upon treatment	ERIC-PCR; qPCR with primers targeting 16S rRNA gene of specific bacterial groups	GS group vs. control: increased: *Lactobacillus* RGS group vs. control: increased: *Bifidobacterium*, *Clostridium* Cluster IV			[120]
*Panax quinquefolius*, radix	ethanolic extract (70%) PQE	2 groups: drinking water; metronidazole-supplemented drinking water; after 7 days, mice received PQE (30 mg/kg/day) (*n* = 3 per group)	male C57BL6 mice (6–8 weeks)	treated for 3 days, fecal samples collected	-	-	HPLC/TOF-MS	compound K detected in feces from mice treated with no antibiotic; undetectable in feces of metronidazole- pretreated mice	[148]
	air-dried American ginseng powder	1 group: 2 g American ginseng powder per day for 7 days (*n* = 6); no control	healthy male volunteers (ages 18–45 years)	day 1 (control) and day 7: feces samples collected	-	-	LC-Q-TOF-MS	16 metabolites in feces: compound K major metabolite; Rk_1_ and Rg_5_, Rk_3_ and Rh_4_, Rg_6_ and F_4_ produced via dehydration	[150]
	air-dried American ginseng powder	1 group: 2 g American ginseng powder in capsules per day for 7 days (*n* = 6), no control	healthy male volunteers (ages 18–45 years); three on Asian diet and three on Western diet	day 1 (control) and day 7: feces samples collected	-	-	LC-Q-TOF-MS	higher relative abundance in Asian diet subjects: ginsenoside Rb1; higher relative abundance in Western diet subjects: compound K, ginsenoside Rh2	[151]
	ethanolic extract (70%) AGE	4 groups: control, azoxymethane/DSS-induced colitis model group, AGE low dose (15 mg/kg/day), AGE high dose (30 mg/kg/day) (*n* = 10 per group)	male A/J mice (6 weeks old with 18–22 g)	treated from day 1 to week 13; fecal samples collected during weeks 1, 2, 5, 8, and 13	terminal-restriction fragment length polymorphism (T-RFLP) with broad-range primers for bacterial domain, followed by 16S rRNA gene NGS Illumina)	AGE vs. model group: increased rel. levels of Firmicutes, decreased rel. levels of *Bacteroidetes* and *Verrucomicrobia*	untargeted GC/TOF-MS	major metabolites: compound K, ginsenoside Rg_3_, and protopanaxadiol	[152]
*Paullinia cupana*, semen	guarana seed powder	3 groups: guarana (0.021 g/kg); caffeine (0.0007 g/kg); saline (1.0 mL/kg) (*n* = 10 per group)	male Wistar rats (250–300 g)	treated for 21 days; fecal samples were collected	16S rRNA gene, NGS (Ion PGM System)	rel. decrease in Bacteroidetes and *Prevotella*, rel. increase in cyanobacteria in guarana group compared to caffeine and saline group; decrease in *Lactobacillus* in caffeine and guarana group	-	-	[156]
	guarana seed powder (Gua)	4 groups: control diet (low-fat, CD); CD + 0.5% Gua; Western diet (WD; high fat); WD + 0.5% Gua (*n* = 12 per group)	male Wistar rats (8 weeks old)	treated for 18 weeks; fecal samples were collected during week 16	V1–V3 region of 16S rRNA gene, NGS (Illumina)	WD + 0.5% Gua compared to WD: increase in *Butyricicoccus* and *Streptococcus*, decrease in *Holdemania*	-	-	[157]
*Polygala tenuifolia*, radix	ethanolic extract (75%) RPE	3 groups: control (saline), 0.5 h group, and 1.5 h group (both RPE 2 g/kg) (*n* = 6 per group)	male Sprague Dawley rats (200 ± 20 g)	treated for 6 days	-	-	targeted UHPLC-Q-TOF-MS	feces of RPE groups: 44 native RPE constituents (3 xanthones, 1 sucrose ester, 9 oligoesters, 33 saponins), and 29 metabolites	[160]
	water extract (100 g radix polygalae powder refluxed at 100 °C with 1 L water) PGW	3 groups: normal diet (ND; *n* = 8), HFD control (HFD-C), HFD- polygala group (HFD-PGW) (PGW dissolved in distilled water orally once daily, dose not given) (*n* = 10 per group)	male ICR mice (4 weeks old)	treated for 5 weeks after model construction, fecal samples collected after 5 weeks treatment	V3–V4 region of 16S rRNA gene, NGS (Illumina)	HFD-PGW group vs. HFD-C group: reduced Bacteroidetes/Firmicutes ratio in HFD-C group mitigated in HFD-PGW group; rel. increase: Proteobacteria, Bacteroidaceae, Rikenellaceae, S24-7, Desulfovibrionaceae, Enterobacteriaceae; rel. decrease: Deferribacteres, Lachnospiraceae, Ruminococcaceae, Peptococcaceae	-	-	[161]
*Polygonatum sibiricum*, radix	ethanolic extract (70%) with a saponin yield of 3.07 ± 0.02 mg/g (PSS)	6 groups: non-diabetic control, diabetic model control (DMC, HFD-streptozotocin induced), metformin-positive control group (MPC), LPT (1 g/kg PSS), MPT (1.5 g/kg PDD), HPT (2 g/kg PSS)	male ICR mice (6 weeks, weight 20 ± 1.5 g)	treated for 5 weeks, fecal samples were collected during week 5	agar plate counting using fecal bacteria selective agars	LPT, MPT, HPT groups vs. DMC group: number of probiotics in the feces increased significantly (*p* < 0.01), especially *Bifidobacterium*; the number of harmful bacteria (*Enterococcus*, Enterobacteriaceae) decreased	-	-	[164]
*Rhodiola rosea*, radix	root extract (SHR-5)	two groups: control group (yeast solution); SHR-5 group (25 mg/mL SHR-5 + yeast solution)	Oregon-R flies	treated throughout the lifespan of the flies; flies were homogenized in PBS for microbiome analyses	V6–V8 region of 16S rRNA gene, NGS (Illumina); bacterial growth plates	SHR-5 group: increase in Acetobacter; decrease in Lactobacillales; SHR-5 decreased the total culturable bacterial load of the fly gut while increasing the overall quantifiable bacterial load	-	-	[167]
*Salvia rosmarinus*, folium	rosemary extract (RE) containing 60% carnosic acid	3 groups: control; chronic restraint stress (CRS) group; CRS + RE (100 mg/kg) (*n* = 12 per group)	male adult ICR mice	treated for 21 days; fecal samples collected (timepoint not indicated)	V1–V3 region of 16S rRNA gene, NGS (Illumina)	CRS+RE group: reversed intestinal microbiota composition of CRS group; rel. increase Firmicutes and *Lactobacillus*; rel. decrease Bacteroidetes and Proteobacteria	-	-	[42]
*Schisandra chinensis*, fructus	total ethanolic extract (95%) (SCE), lignan fraction (SCL), polysaccharide fraction (SCPS), volatile oil (SCVO)	6 groups: control, lipopolysaccharide (LPS)-induced inflammation, SCE (1.2 g/kg) + LPS, SCL (500 mg/kg BW) + LPS, SCPS (300 mg/kg) + LPS, SCVO (150 mg/kg BW) + LPS (*n* = 10 per group)	C57BL/6 mice (18–22 g)	treated for 14 days; fecal samples collected after behavioral tests	V3–V4 region of 16S rRNA gene, NGS (Illumina)	SCE and SCL-treated group: LPS-induced increase in Bacteroidetes and decrease in Firmicutes alleviated rel. increase: *Lactobacillus*; rel. decrease: *Bacteroides*	SCFA analysis by GC-MSTQ8040	SCE and SCL-treated group: increased levels of butyric acid and propionic acid	[173]
	dried, powdered fruits (SC); wine- processed fruits (WSC); main SC and WSC constituent: lignans	4 groups: control (0.9% saline); chronic unpredictable stress procedure (CUSP) group; CUSP + SC (280 mg/kg BW); CUSP + WSC (280 mg/kg BW) (*n* = 6 per group)	male Sprague Dawley rats (180–220 g)	treated for 5 weeks; fresh fecal samples collected on day 30	V3–V4 region of 16S rRNA gene, NGS; (Illumina)	CUSP+SC/WSC vs. CUSP: increased rel. abundance of Lachnospiraceae; rel. decrease in *Bacteroides*	lactate analysis in the intestine by ELISA	reduction: D- and L-lactate	[172]
	water extract (SCW)	two groups: placebo (*n* = 15); SCW (*n* = 13) 2 pouches in a day, equivalent to 6.7 g of dried *S. chinensis* fruits	female obese volunteers BMI ≥ 25 kg/m^2^	feces samples collected at the beginning and the end of treatment	denaturing gradient gel electrophoresis; qPCR with specific primers	SCF group vs. placebo: increase: *Akkermansia*, *Roseburia*, *Bacteroides*, *Prevotella*, *Bifidobacterium*; decrease: *Ruminococcus*	-		[174]
	*S. chinensis* polysaccharide extract (total carbohydrate content: 94.9%) (SCP)	4 groups: normal control (saline), model group (DSS-induced colitis), DSS+ positive control (salazosulfapyridine), DSS + SCP (8.0 g/kg BW) (*n* = 8 per group)	male C57BL/6J mice (20 ± 2 g, 8–10 weeks old)	treated for 3 weeks	16S rRNA gene, NGS (Illumina)	SCP vs. DSS group: Firmicutes, Proteobacteria, and Bacteroidetes returned to normal relative abundances; rel. increase: *Alloprevotella*, Saccharibacteria, Bacteroidetes Bacteroidales_S24_7_group family; rel. decrease: *Anaerotruncus*, Firmicutes	SCFA analysis by GC-MS	SCP vs. DSS group: recovery/increase in propionic acid, butyric acid, valeric acid	[175]
*Trigonella foenum-graecum*, semen	ground seeds (2% of the diet by weight) (FS)	4 groups: HFD; HFD + FG; control diet (CD); CD + FG (*n* = 20 per group)	male C57BL/6J mice (9 weeks old)	treated for 16 weeks; fecal samples collected after euthanasia	V4 region of 16S rRNA gene, NGS (Illumina)	CD ± FS and HFD ± FS: shifts in alpha and beta diversity compared to non-FS groups; diversity and significantly increased alpha diversity; FS mitigated dysbiotic effects of HFD	-	-	[177]
	fenugreek seeds (28% galactomannan and 0.672% apigenin-7-glycoside) FS	2 groups: control (*n* = 11); FS (*n* = 10, 1.5 g fenugreek seeds/kg BW)	male castrated piglets (Duroc × Piétrain; 8.26 kg)	treated for 28 days; stomach, distal jejunum, ileum, cecum, and colon contents removed after sacrifice	qPCR with specific primers	increase: *Lactobacillus* group, *L. johnsonii*, *Clostridium* cluster I, *L. reuteri* decrease: *Escherichia/Hafnia/Shigella* group *Clostridium* cluster YIV remained stable	lactate (HPLC), SCFAs (GC-FID)	FS vs. control group: increased colonic butyric acid levels; increased L-lactic acid levels in the small intestinal digesta	[178]
*Vitis vinifera*, fructus	lyophilized table grape mixture of red-, green-, and black-seeded and seedless grapes (G)	5 groups: low fat (LF; 10% of energy from fat); high fat (HF; 34% of energy from fat) plus 3% G (*w*/*w*; HF-3G); HF plus 3% sugar (*w*/*w*; HF-3S); HF plus 5% G (HF-5G); HF plus 5% sugar (HF-5S) (*n* = 10 per group)	male C57BL/6J mice (4 weeks old)	treated for 11 weeks; colonic mucosa and digesta from duodenum, jejunum, cecum, proximal and distal colon collected after sacrifice	qPCR with primers targeting 16S rRNA gene of specific bacterial genera; V3–V4 region of 16S rRNA, Illumina sequencing	decreased alpha diversity in HF-5G and HF-5S group compared to HF-3G group; increase in *Allobaculum* in LF and HF-3G group; tendency to increase in *Akkermansia muciniphila* in HF-3G and HF-5G group; decrease in *Desulfobacter* spp. in HF-3G group	-	-	[197]
	phenolic compound-rich grape pomace extract (70% ethanol; 920 mg/g phenolic compounds) (PC)	5 groups: PC 2.5 (2.5 mg/kgBW/d); PC 5 (5 mg/kg BW/d); PC 10 (10 mg/kg BW/d); PC 20 (20 mg/kg/d); control group (0.1% DMSO) (*n* = 6 per group)	male adult Wistar rats (2 months old)	treated for 14 months; fecal samples collected at baseline, and after 6 and 14 months of treatment	qPCR with primers targeting 16S rRNA gene of specific bacterial genera and universal primer for total bacteria	increase in *Bifidobacterium* in PC 2.5 and PC 5 groups after 6 and 14 months compared to control and young rats; PC (all groups) abolished increase in *Clostridium* (cluster 1) after 14 months occurring in control	-	-	[194]
	grape antioxidant dietary fiber (GADF)	2 groups: control diet; GADF diet (50 g/kg) (*n* = 10 per group)	male Wistar rats (body weight of 215 ± 2 g)	treated for 4 weeks; cecal content collected after sacrifice	qPCR with primers targeting 16S rRNA gene of specific bacterial genera	GADF group: increase: *Lactobacillus* spp. decrease: *Bifidobacterium* spp.	-	-	[195]
	grape seed and grape marc meal extract (GSGME)	3 groups: control group (basal diet BD); GSGME group (BD with 1% GSGME) (*n* = 16 per group)	crossbreed pigs (5 weeks old)	treated for 4 weeks; fecal samples collected after sacrifice	qPCR with primers targeting 16S rRNA gene of specific bacterial genera	decrease in *Streptococcus* in GSGME group	volatile fatty acid analysis by GC with FI detector	Decrease in acetic acid, propionic acid, and valeric acid in GSGME group	[196]
	grape extract (GE)	3 groups: standard diet (LFD, 3.85 kcal g^−1^, 10% energy from fat); high-fat +high-fructose diet (HFFD, 4.73 kcal g^−1^, 22% fructose + 22% lard); HFFD + 1% *w*/*w* GE diet (HFFD + GE) (*n* = 12 per group)	male C57BL/6Cnc mice (4 weeks old)	treated for 13 weeks; fecal samples were collected after sacrifice	V3–V4 region of 16S rRNA gene, NGS	GE group: increased gut microbiota diversity, Firmicutes/Bacteroidetes ratio, rel. increase in *Verrucomicrobia*, *Bifidobacteria*, *Akkermansia*, *Clostridia*; rel. decrease in *Bacteroidetes*, *Proteobacteria*, *Desulfovibrio*, and *Bacteroides*	-	-	[199]
	lyophilized table grape mixture (red-, green-, and black-seeded and seedless) (GP); extractable polyphenol-rich fraction (EP) (180 mg/g total phenolics); nonextractable, polyphenol-poor fraction (NEP) (10.5 mg/g total phenolics)	6 groups: low fat (LF; 10% of energy from fat); high fat (HF; 44% of energy from fat); HF plus extractable polyphenol-rich fraction (HF-EP); HF plus nonextractable, polyphenol-poor fraction (HF-NEP); HF plus extractable and nonextractable polyphenol fraction (HF-EP + NEP); HF plus 5% powdered grapes (HF-GP) (*n* = 10 per group)	male C57BL/6J mice (4 weeks old)	treated for 16 weeks; cecal mucosa and digesta samples collected after sacrifice	V4–V5 region of 16S rRNA gene, NGS (Illumina) of cecal mucosa samples	HF-GP vs. HF control: rel. increase in microbiota diversity compared to HF control group *HF-EP* vs. *HF-control*: rel. increase in Lachnospiraceae HF-NEP vs. HF-control: rel. increase in Coprococcus HF-EP+NEP vs. HF-control: rel. increase in Lachnospiraceae and Coprococcus; rel. decrease in Ruminococcus and Mogibacteriaceae	SCFA analysis in cecal digesta by GC-MS-MS	HF-GP vs. HF-EP + NEP group: increase in the SCFAs acetate, propionate, and butyrate HF-EP + NEP vs. HF control group: decrease in cecal acetate	[198]
	sun-dried raisins	1 group: three servings per day of 28.3 g raisins (90 cal, 2 g dietary fiber) (*n* = 13)	healthy volunteers (ages 18–59 years)	treated for 2 weeks; fecal samples collected before the start of raisin consumption, on day 7 and day 14	V1–V2 region of 16S rRNA gene, NGS (Illumina)	weeks 1 and 2 vs. day 0: rel. increase in Ruminococcaceae; *Faecalibacterium prausnitzii*, and *Bacteroidetes longum* rel. decrease in *Bifidobacterium* spp., *Klebsiella* spp., *Prevotella* spp.	-	-	[192]
	red grape pomace (GP) extract (Eminol^®^)	1 group: two capsules of GP extract per day (1400 mg GP/day) (*n* = 10)	healthy female volunteers (ages 25–65 years; BMI < 25 kg/m^2^)	treated for 21 days; fecal samples collected after washout period, on day 14 and on day 21 of GP consumption	qPCR with primers targeting specific bacterial genera	no change in the intestinal microbiota composition	phenolic metabolite analysis by UPLC-ESI-MS/MS; short- and medium-chain fatty acid analysis by SPME-GCMS	day 0 vs. day 7 or 14: SCFA: increase in total SCFAs and propionic acid (14 and 21 days); increase in acetic acid (14 days) MCFA: decrease in pentanoic, hexanoic, and octanoic acids; fecal phenolic metabolites: increase in 3-(4′-hydroxyphenyl)-propionic acid	[200]
*Vitis vinifera*, semen	grape seed tannins: monomer fraction (GSM); polymer fraction (GSP)	3 groups: control group (standard diet), GSM group (standard diet + GSM 71 mg/kg diet), GSP (standard diet + GSP, 71 mg/kg diet) (*n* = 6 per group)	male Sprague Dawley rats (145 g)	treated for 12 weeks; cecal contents were collected after sacrifice	-	-	cecal volatile fatty acid (SCFA) analysis by GC	GSP vs. control: increase in total VFAs, acetate, propionate, and butyrate *GMP* vs. *control:* increase in acetate, decrease in butyrate	[184]
	grape seed extract (GSE)	1 group: standard diet (SD, 2 kg per day), treatment diet (SD plus 1% *w*/*w* GSE) (*n* = 6)	crossbred female pigs (130–150 kg)	duration 12 days; SD for 3 days, SD+GSE for 6 days, post-treatment SD for 3 days; fecal samples collected daily	V3–V4 region of 16S rRNA gene NGS (Illumina)	before vs. during GSE: increase in Lachnospiraceae, unclassified *Clostridales*, *Lactobacillus*, and *Ruminococcus*	phenolic metabolite analysis by HPLC-MS	before vs. during GSE: increase in 4-hydroxyphenylvaleric acid and 3-hydroxybenzoic acid	[185]
	grape seed meal (GSM)	4 groups: control group (standard diet, SD); AFB1 group (SD + 320 µg/kg aflatoxin B1, AFB1); GSM group (SD+ 8% GSM); AFB1 + GSM group (SD + 32 µg/kg AFB1 + 8% GSM) (*n* = 6 per group)	healthy weaned crossbred TOPIGS-40 hybrid piglets (9.13 ± 0.03 kg)	treated for 30 days; colon contents collected after sacrifice	V3–V4 region of 16S rRNA gene NGS	GS vs. control: rel. increase in Bacteroidetes, Proteobacteria, *Prevotella*, *Megasphaera*, Clostridiales, and *Anaerovibrio*; rel. decrease in Firmicutes, *Lactobacillus*, and Lachnospiraceae	-	-	[186]
	grape seed meal (GSM)	4 groups: control group (standard diet, SD); DSS colitis group (SD + DSS 1 g/kg BW); GSM group (SD + 8% GSM); DSS+GSM group (SD + 8% GSM + DSS 1 g/kg BW) (*n* = 5–6 per group)	weaned crossbred TOPIGS-40 hybrid piglets (9.13 ± 0.03 kg)	treated for 30 days; descending colon contents collected after sacrifice	V3–V4 region of 16S rRNA gene NGS (Illumina)	rel. increase in Proteobacteria and rel. decrease in Lactobacillus in DSS, GSM, and DSS + GMS group; rel. increase in *Megasphaera* and *Anaerovibrio* in GSM and DSS+GSM groups; rel. decrease in *Roseburia* in GSM and DSS + GSM groups	SCFA analysis by GC-FID	increase in butyric acid and valeric acid, and decrease in acetic acid by GSM	[187]
	GSE Leucoselect^®^ (proanthocyanidin content >80%)	3 groups: sham-operated group (standard diet, SD); OVX group (SD); OVX + GSE group (GSE diet, 10 g GSE/5 kg diet) (*n* = 5 per group)	female C57BL/6J mice (7 weeks old)	treated for 8 weeks; fecal samples were collected 8 weeks after surgery	qPCR with group-specific primers targeting 16S rRNA of total bacteria, Firmicutes, and Bacteroidetes	OVX + GSE vs. OVX group: increase in Bacteroidetes; decrease in Firmicutes and Firmicutes/Bacteroidetes ratio	-	-	[188]
	GSE Vitaflavan^®^ (procyanidin content 75.6%)	4 groups: control LFD (10% kcal from fat, CD); HFD (45% kcal from fat); HFD + 0.07 g GSE/4057 kcal (HF10); HFD + 0.70 g GSE/4057 kcal (HF100) (*n* = 8 per group)	male C57BL/6J mice (9 weeks old)	treated for 16 weeks; small intestine, cecum, and colonic tissue collected after sacrifice	V4 region of 16S rRNA gene NGS (Illumina) of mucosal-adherent metabolically active bacteria (results converted to 16S cDNA values; HF 100 group not analyzed)	HF10 group vs. HFD: small intestine: decrease in Firmicutes, *Bacteroides*-*Prevotella* spp., and *Parabacteroides* spp.; increase in Bacteroidetes and *Bifidobacterium* spp.	-	-	[189]
	proanthocyanidin-rich GSE	1 group, 3 treatments: 0.5 g GSE/day (0.19 g/day/subject as proanthocyanidin); 0.5 g green tea extract/day; 0.5 g champignon extract/day	9 healthy male adults (ages 37–42 years)	duration 10 weeks; 6 periods: 14-day washout period, three 14-day administration periods interrupted by two 14-day washout periods; fecal samples collected on days 0, 2, 7, and 14 of administration	bacterial plate counting	GSE, day 14 vs. day 0: increase in *Bifidobacterium*; tendency to decrease in Enterobacteriaceae	fecal putrefactive product analysis by GC; ammonium analysis by HPLC	GSE, day 14 vs. day 0: tendency to decrease in skatol, indole, 4-ethylphenol, p-cresol, phenol, and ammonia after grape seed extract administration	[190]

## Abbreviations

AAD—antibiotic-associated diarrhea; AC—ascending colon; BBB—blood–brain barrier; BCFA—branched-chain fatty acid; CNS—central nervous system; CRF –corticotropine releasing factor; CUSP—chronic unpredictable stress procedure; DSS—dextran sodium sulfate; EC—enterochromaffin cell; DC—descending colon; EEC—enteroendocrine cell; ENS—enteric nervous system; ERIC-PCR—enterobacterial repetitive intergenic consensus PCR; FISH—fluorescent in situ hybridization; FMT—fecal microbiota transplant; GABA—gamma-aminobutyric acid; GAE—gallic acid equivalent; GI—gastrointestinal; HFD—high-fat diet; HPA—hypothalamic–pituitary–adrenal; LEfSe: linear discriminant analysis effect size; LPS—lipopolysaccharide; MDD—major depressive disorder; MGBA—microbiome–gut–brain axis; NF-κB—nuclear factor kappa B; NGS—next-generation sequencing; SCFA—short-chain fatty acid; TJPs—tight junction proteins; TNF—tumor necrosis factor.
